# Genome-wide host responses against infectious laryngotracheitis virus vaccine infection in chicken embryo lung cells

**DOI:** 10.1186/1471-2164-13-143

**Published:** 2012-04-24

**Authors:** Jeongyoon Lee, Walter G Bottje, Byung-Whi Kong

**Affiliations:** 1 Department of Poultry Science, Division of Agriculture, POSC O-404, 1260 West Maple, Fayetteville, AR 72701, USA; 2 Cell and Molecular Biology Graduate Program, University of Arkansas, Fayetteville, AR 72701, USA

## Abstract

**Background:**

Infectious laryngotracheitis virus (ILTV; gallid herpesvirus 1) infection causes high mortality and huge economic losses in the poultry industry. To protect chickens against ILTV infection, chicken-embryo origin (CEO) and tissue-culture origin (TCO) vaccines have been used. However, the transmission of vaccine ILTV from vaccinated- to unvaccinated chickens can cause severe respiratory disease. Previously, host cell responses against virulent ILTV infections were determined by microarray analysis. In this study, a microarray analysis was performed to understand host-vaccine ILTV interactions at the host gene transcription level.

**Results:**

The 44 K chicken oligo microarrays were used, and the results were compared to those found in virulent ILTV infection. Total RNAs extracted from vaccine ILTV infected chicken embryo lung cells at 1, 2, 3 and 4 days post infection (dpi), compared to 0 dpi, were subjected to microarray assay using the two color hybridization method. Data analysis using JMP Genomics 5.0 and the Ingenuity Pathway Analysis (IPA) program showed that 213 differentially expressed genes could be grouped into a number of functional categories including tissue development, cellular growth and proliferation, cellular movement, and inflammatory responses. Moreover, 10 possible gene networks were created by the IPA program to show intermolecular connections. Interestingly, of 213 differentially expressed genes, BMP2, C8orf79, F10, and NPY were expressed distinctly in vaccine ILTV infection when compared to virulent ILTV infection.

**Conclusions:**

Comprehensive knowledge of gene expression and biological functionalities of host factors during vaccine ILTV infection can provide insight into host cellular defense mechanisms compared to those of virulent ILTV.

## Background

Avian infectious laryngotracheitis virus (ILTV), named as a Gallid herpesvirus 1, is a member of the Iltovirus genus, Alphaherpesvirinae subfamily, and Herpesviridae family. ILTV has a linearized dsDNA genome of approximately 150 kb in size which contains unique long (UL) and unique short (US) sequences flanked by inverted repeat (IR) and terminal repeat (TR) sequences [1,2]. The genome encodes 80 predicted viral protein open reading frames (ORFs). ILTV infection causes respiratory disease symptoms in chickens, pheasants, partridges, and peafowl [3,4]. Clinical signs include extension of the neck, gasping, gurgling, rattling, and coughing of clotted blood [5]. ILTV usually causes a reduction in egg production and variable mortality ranging from 5 to 70%, and can cause severe economic losses in the poultry industry [6].

Two types of commercial live attenuated vaccines, chicken embryo origin (CEO) and tissue culture origin (TCO), have been widely used to immunize chicken flocks against ILTV [6,7]. However, it was found that live vaccines infect the nervous system similarly to virulent ILTV infections, and could possibly induce vaccinal laryngotracheitis (VLT) by transmission to unvaccinated birds [8-10]. Moreover, global ILTV outbreaks are mostly associated with CEO vaccines [11-13] and the genomic- and antigenic characteristics between virulent and vaccine ILTV are very similar [6].

Microarray analysis has become popular, along with the recent development of a RNA-seq (RNA sequencing) technique using next-generation sequencing, to analyze comprehensive gene expression in different biological conditions. Microarrays have been performed intensively to investigate host gene transcriptional responses to infection by various viruses such as hepatitis C virus (HCV) [14], rice dwarf virus (RDV) [15], influenza virus [16], herpesvirus saimiri (HVS) [17], human immunodeficiency virus (HIV) [18,19], Japanese encephalitis virus (JEV) [20,21], chicken anemia virus (CAV) [22], human cytomegalovirus (hCMV) [23], Epstein-Barr virus (EBV) [24], infectious laryngotracheitis virus (ILTV) [25], varicella-zoster virus (VZV) [26], alphaherpesvirus [27], Marek's disease virus (MDV) [28], herpes simplex virus type 1 (HSV-1) [29], even in vaccine strains including recombinant flavivirus [30], west nile/dengue 4 virus [31], and dengue virus [32].

Previously, we studied the differential gene expression of host responses against virulent ILTV infection in cultured primary chicken embryo lung cells using microarray analysis [25]. To compare and contrast host responses to infection of vaccine ILTV to those of virulent ILTV infection, primary chicken embryo lung cells were infected with live attenuated CEO vaccines and host gene expression during a four day period post infection was determined using 44 K chicken oligo microarrays in the present study.

## Results and discussion

### Profiling of differentially expressed host genes in vaccine ILTV infection

**Figure 1:**
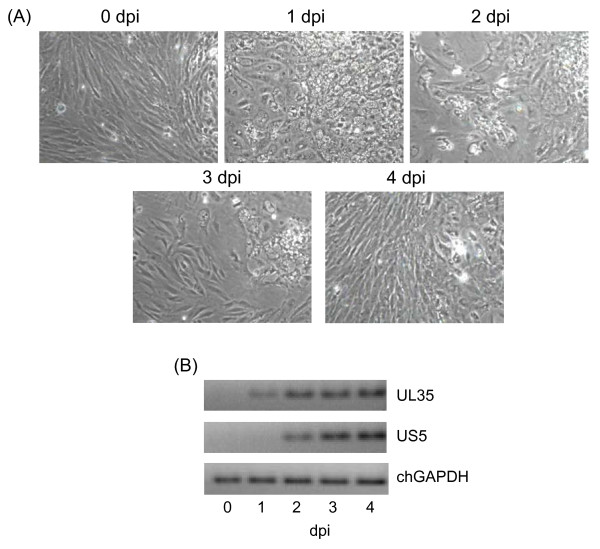
**Morphology of chicken embryo lung cells infected by vaccine ILTV and the expression of ILTV genes**. (A) Cell morphology and cytopathic effect development at 0, 1, 2, 3 and 4 dpi are displayed by phase-contrast microscopic images at 200 × magnification. (B) ILTV gene expression for UL35 and US5 was determined by RT-PCR. Chicken GAPDH (chGAPDH) was used as an endogenous control.

Primary chicken embryo lung cells at passage 1 were infected with 3 vaccination doses of a live fowl laryngotracheitis vaccine, which is widely used in the poultry industry. The infected cells were subjected to analysis for cytopathic effects and virus infection validation at 1, 2, 3 and 4 days post infection (dpi). Although weak cytopathic effects (e.g. cell rounding, aggregation and syncytia) were observed at certain locations of plates at 1 and 2 dpi, infected cells began to recover by 3 dpi, and no cytopathic effects were observed at 4 dpi (Figure 1A). To verify the infection of vaccine ILTV, the expression of ILTV viral RNA was determined and genes of UL35 encoding a small capsid protein and US5 encoding an envelop glycoprotein J (gJ) were shown to progressively increase their expression post infection though US5 expression began to be detected from 2 dpi (Figure 1B).

In the 44 K microarray assay, two approaches to avoid possible hidden dye effects were used: the use of RNA Spike-in controls synthesized from the Adenovirus E1A transcriptomes containing different concentrations of dye in each set [33] and the dye-swap in two of four total replicates. No significant dye effects were detected in all microarray slides (data not shown). Data analysis using one-way ANOVA with the JMP Genomics 5.0 and a 2 fold or greater cut off value revealed that 933 genes were differentially expressed at 4 different dpi time points following vaccine ILTV infection (Additional file 1). When these 933 differentially expressed genes were subjected to the bioinformatics study using Ingenuity Pathways Analysis (IPA, Ingenuity^® ^Systems, http://www.ingenuity.com) to generate the gene networks and functional annotations, 213 genes were recognized as mapped IDs (functionally known genes) by the IPA program (Additional file 2) and subjected to further bioinformatics analysis.

### Quantitative reverse transcription-PCR (qPCR)

To validate the microarray results, expression of 18 of the 933 differentially expressed genes was subjected to qPCR using the same RNA samples as those used in the microarray, and gene specific primer sets (Tables 1, 2). Of the 18 genes tested, the expression pattern for 12 genes completely matched the microarray data at four dpi time points. The expression pattern for the remaining 6 genes also qualitatively matched to microarray data, though they were not quantitatively matched (Table 2). With the comparison of the spike-in controls, qPCR results indicated that the microarray data in this experiment were valid to determine host gene expression responses against vaccine ILTV infection.

**Table 1:** Primers for qPCR

GenBank	Forward Primer	Reverse Primer
AB109635	GGCACCAACTTGCTACCACA	GCTGCAAGAGCTGCCATTAG

AF505881	CCAGCTACATCTCCCACCTG	TCTGTTTGGGCTGGGAGTTC

BX932962	GTACAACCACGTTGGGCAGA	CTCTCGCGTTTCCTTTGAGG

BX931297	TTGTGGCCTACCCCAGATGT	GGATGGGACTTTCCAGAGCA

BX931599	CTGTTTCCTGACCGCAGTTC	AGCACAAACTCCGCCATTTT

BX933478	CCTGTGCAAGGTGTCCAGTG	CCCAATGGCCATACAGTTCA

BX933728	GCAAAGCACCATCCCAAATA	TAT TGAGGCGCTGACTCCTG

BX933888	CTGGGATCCCTCCAGAGCTA	CCATTCACTGGAGCACCAAA

BX935456	CGAGGCCATCAACTTCCTTC	TCCACATGACGCACATACCC

BX936211	CCAGCTGTCCTCCTTGGAAT	AGGGAGAGGAAGACGTGCTG

CR352775	GGCCTTGAATGACCATCATGT	AACAGCAGCAGGAACAGTGC

CR385566	CGCGCTCTACGACTACATGC	CTGGGTGGTGATCTCGGTCT

D87992	TGCAGCACTGAGACCTGGAT	CAGTTGCTGCGGATGAAGTC

M60853	TTTTGGCTACCAGTCCAGCA	TTCGCAAGTGTTCCCCAGTA

M64990	TGCTCCCCTGAGTACTGGAA	GCCTCTGTGGGTTCAGGATT

M80584	TCCCACTGAGCAGCTTCTGTA	CCAGAGAGATATCCGCAGCA

M87294	GGTGCTGACTTTCGCCTTGT	GCCTGGTGATGAGGTTGATG

X87609	CCACCTGAGAAAAGCGACCT	ACATCGACCTCTGCCAACCT

**Table 2:** Validation of gene expression between microarray and qPCR

GenBank	Symbol	Fold Change (Microarray/qPCR)			
		
		Day 1	Day 2	Day 3	Day 4
BX932962	SLC37A2	1.2/1.3	2.1/2.6	3.3/3.7	3.9/4.5

M60853	THBS2	1.5/1.8	2.0/2.5	2.8/2.8	3.3/3.3

BX933888	C1QTNF3	-0.2/0.1	1.2/2.0	1.9/2.5	2.7/3.4

BX933728	CAPSL	2.3/3.0	2.6/3.7	3.2/4.3	3.5/4.4

BX931297	CYTL1	1.0/1.7	1.4/2.8	2.7/4.2	2.9/4.4

CR352775	ALDOB	0.7/1.1	1.0/1.7	2.1/2.2	2.7/2.5

BX933478	MXRA5	0.8/1.5	1.7/3.0	2.5/3.7	3.0/4.4

BX935456	EGLN3	-2.7/-2.0	-2.3/-1.9	-1.7/-1.4	-1.3/-0.9

BX931599	VIPR2	1.7/2.0	2.3/2.9	2.9/3.6	3.1/5.1

CR385566	CLEC3B	0.2/1.0	0.4/2.2	1.3/2.4	1.4/3.1

M87294	NPY	-0.4/-0.3	-0.9/-0.6	-1.5/-0. 9	-1.7/-1.3

M80584	LUM	1.2/1.6	1. 5/2.3	1.9/2.5	2.5/3.2

M64990	PTGS2	-0.6/-0.6	-0.8/-0.3	-0.2/-0.4	0.7/1.2

AB109635	HMGCR	0.4/0.4	0.2/0.6	-0.5/-0.3	-0.9/-0.6

BX936211	TMEM116	0.0/0.0	-0.3/0.3	-0.3/-0.1	-1.3/-0.7

X87609	FST	1.3/1.0	0.6/1.2	0.6/0.6	-0.3/0.2

D87992	ANPEP	-0.1/-1.2	-0.5/0.5	-0.6/0.4	-1.4/-0.2

AF505881	SCX	1.1/0.2	1.4/-0.6	1.8/0.1	2.3/-0.6

The gene expression levels of microarray were presented by log_2 _fold changes, whereas those of qPCR were indicated by -ΔΔCt that are comparable to the log_2 _fold change values in microarray

### Biological functions of differentially expressed genes

**Figure 2:**
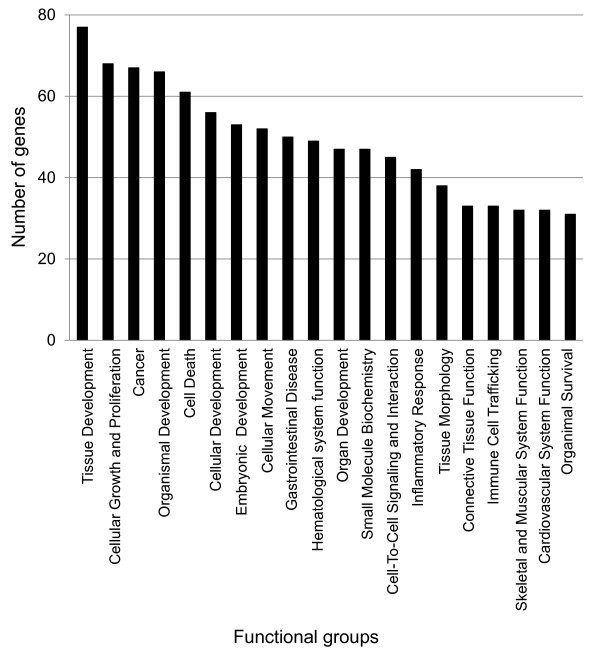
**Functional groups of differentially expressed genes**. Out of 75 functional groups, the top 20 groups considered based on gene number were displayed.

Using the IPA program, the subset of 213 mapped genes were categorized into 75 biologically functional groups, and the top 20 groups associated with a greatest number of genes, are shown in Figure 2. The 20 functional groups are mostly related to tissue development, cellular growth and proliferation, organismal development, cell death, cellular development, cellular movement, and inflammatory responses.

### Top 10 differentially expressed genes

**Figure 3:**
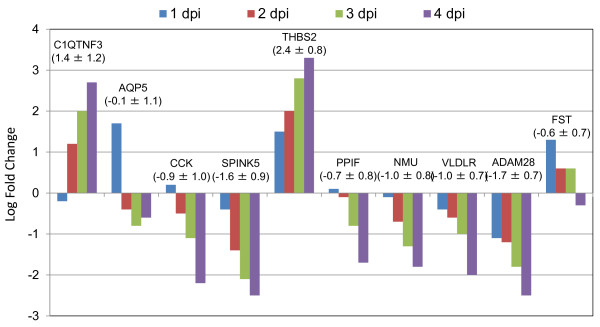
**The 10 most differentially expressed genes in ILTV vaccine infection**. The blue, red, green and purple bars represent 1 dpi, 2 dpi, 3 dpi, and 4 dpi for vaccine ILTV infections, respectively. Mean ± standard deviation value of log_2 _fold change among four dpi time points are indicated under the gene name.

Of 213 genes with known functions with mapped IDs, the top 10 most differentially expressed genes in ILTV vaccine infection were selected based on differences of standard deviation (SD) among all four dpi time points (Figure 3). The 10 most differentially expressed genes are involved in functions of reduced inflammatory responses, stimulation of cell proliferation, suppression of apoptosis, and promotion of cell-to-cell interactions. The general function and possible roles of these differentially expressed genes during ILTV infection are outlined in Table 3.

**Table 3:** Functions of the 10 most differentially expressed genes in ILTV vaccine infection

Symbol	Functions
C1QTNF3 (CTRP3)	• C1q and tumor necrosis factor related protein 3, a. k. a. CTRP3. • Functions are described in the Result and Discussion.

AQP5	• Aquaporin 5, a water channel protein. • Functions are described in the Result and Discussion.

CCK	• Cholecystokinin • Functions are described in the Result and Discussion.

SPINK5	• Serine peptidase inhibitor, Kazal type 5 or chicken ovomucoid • Lymph-epithelial Kazal-type-related inhibitor (LEKTI). • Suppresses cellular functions related to inflammation in human primary keratinocytes (HK) [34]. • Down-regulation of SPINK5 at all dpi may support the reduced cell death caused by vaccine ILTV infection.

THBS2	• Thrombospondin 2, a potent inhibitor of tumor growth and angiogenesis and a matricellular glycoprotein which mediates cell-to-cell interaction [35]. • Functions in angiogenesis in patients with early-stage non-small cell lung cancer [36], and wound healing and development of exuberant granulation tissue in horses [37]. • Up-regulation of THBS2 in vaccine ILTV infection may function in virus spread in infected cells.

PPIF	• Peptidylprolyl isomerase F, one of the peptidyl-prolyl cis-trans isomerase (PPIase) family proteins and a member of the mitochondrial permeability transition (PT) pore in the inner mitochondrial membrane. • Stimulates the cis-trans isomerization of proline imidic peptide bonds in oligopeptides and accelerate the folding of proteins [38,39]. • Apoptosis and necrosis of cells were induced by the activation of the PT pore [40-42]. • Down-regulation of PPIF genes in vaccine ILTV infection may play a role in cell death and recovery of cells.

VLDVR	• Very low density lipoprotein/vitellogenin receptor • Binds to baculovirus surface membrane to inhibit ligand-receptor interaction in viral infection of HeLa cells [43]. • The meaning of down-regulation of VLDLR in ILTV vaccine infection in addition to in other herpesvirus infection, is unknown.

NMU	• Neuromedin U, a multifunctional neuropeptide • Functions in conditions of pain and stress, the metabolism and homeostasis of feeding and energy in body, inflammatory diseases, smooth muscle contraction, and the control of blood flow and pressure [44,45]. • Induces early-phase inflammation through the degranulation in mast cells in which NMU-R1 is highly expressed [46]. • Acts as an inflammatory mediator via the acceleration of IL-6 production in macrophages [47]. • Down-regulation of NMU may represent the inhibition of cellular factors associated with inflammation.

ADAM28	• A disintegrin and metalloproteinase (ADAM) domain 28. • Functions in cell-to-cell and cell-to-matrix interaction on the cell surface for cancer cell proliferation, invasion and metastasis [48,49]. • Up-regulated at carcinoma cells and functions the proliferation and progression of human lung and breast cancer cells [50,51]. • Acts as an inhibitor against human dental pulp stem cells (HDPSCs) proliferation and an inducer of apoptosis of HDPSCs through the stimulation of alkaline phosphatase (ALP) secretion and dentin sialophosphoprotein (DSPP) [52]. • Degrades Insulin-like growth factor (IGF) binding protein 3 (IGFBP3) [53]. • The decreased expression of ADAM28 in vaccine ILTV infection, may suppress the active induction of apoptosis.

FST	• Follistatin • Inhibits follicle-stimulating hormone [54]. • Binds and neutralizes activin, a paracrine hormone of TGF-β superfamily, which is related to the regulation of cell proliferation, apoptosis, and carcinogenesis [55,56]. • A member of fibrotic and wound healing response genes and cellular proliferation genes and plays a role in muscle growth and strength in nonhuman primates and liver proliferation. Moreover, the small plaque mutant of VZV down-regulates FST [57]. • Up-regulation of FST at early phase (1 dpi) of vaccine ILTV infection may play a role in the initiation of cytopathic effect.

The C1q and tumor necrosis factor related protein 3 [C1QTNF3; also known as (a. k. a.) CTRP3] is known to stimulate ERK1/2 and p38 MAPK [58]. C1QTNF3 expression was elevated from 2 to 4 dpi (Figure 3). In addition, cartonectin (an adipokine of the CTRP3 family) helps reduce inflammation by suppressing IL6 and TNF production and NF-κB signaling [59-61]. IL6, one of several cytomegalovirus secreted cytokines, enhances the survival of endothelial cells by blocking caspase 3 and caspase 7-mediated apoptosis [62]. CTRP3 could also accelerate embryonic growth and may contribute to a high feed efficiency phenotype in broilers [63]. Thus, the continuous increase of CTRP3 expression from 2 to 4 dpi during ILTV vaccine infection suggests the suppression of cellular factors related to inflammation and the promotion of cell recovery after the initial cytopathic effect that was exhibited in embryonic lung cells on 1dpi.

Aquaporin 5 (AQP5) expression, a water channel protein and part of the small integral membrane protein family, was elevated on 1 dpi, but decreased at 2- and 4- dpi (Figure 3). A deficiency AQP5 was shown to exacerbate lung injury by the infection of *Pseudomonas aeruginosa *[64]. Down-regulation of AQP5 increases proliferation and migration of human corneal epithelial (CEP117) cell line [65]. Adenovirus infection to mouse lung caused the down-regulation of AQP5 gene expression [66]. Thus, the slight down-regulation of AQP5 gene expression in vaccine ILTV infection in from 2 to 4 dpi may be associated with the reduced cytopathic effect and cell recovery.

Cholecystokinin (CCK) was down-regulated in lung cells between 2 and 4 dpi (Figure 3). CCK is a peptide hormone of the gastrointestinal system that is important in fat digestion [67]. CCK induces apoptosis by stimulating death signaling pathways in rat pancreatic acinar cells, including caspase activation, cytochrome C release, and mitochondrial depolarization [68]. Thus, the down-regulation of CCK expression in vaccine ILTV infected cells may suppress apoptosis, resulting in weak cytopathic effects.

### Gene networks

Network analysis by IPA was used to draw connections between interacting focus molecules. Of the 10 networks that were generated, the top 4 networks were identical among all four dpi, which may be due to the fact that the algorithms of the IPA program generate a network by considering fold change values and p-values of focus molecules in addition to their biological functions. The lists, top functions, and the most focused molecules of all four networks are shown in Additional file 3, and the drawings of interacting molecules in each network during the time course of all dpi are displayed in Additional file 4.

**Figure 4:**
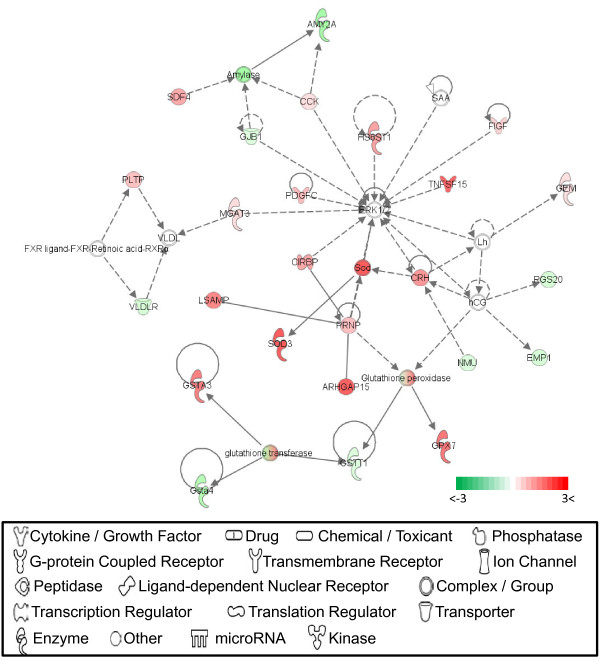
**Network #1 at 1dpi**. Symbols of functions for each molecule used to generate the molecular network are displayed. The green indicates down-regulation, while the red depicts up-regulation. White symbols indicate neighboring genes that are functionally associated but not included in the differentially expressed gene list. The intensity of color represents the average of log_2 _fold change in a given population. The numbers below the color change bar denote log_2 _values. Symbols for each molecule are presented according to molecular functions and type of interactions.

The most interactive network (network #1) at 1 dpi is presented in Figure 4. Similar differential expression levels for focus molecules in network #1 are shown at all four dpi during ILTV vaccine infection and the top functions related to genes in this network include free radical scavenging, lipid metabolism, and small molecule biochemistry. Several secreting proteins such as FIGF [c-fos induced growth factor; a.k.a vascular endothelial growth factor D (VEGF-D)], PDGFC (platelet derived growth factor C), TNFSF15 (tumor necrosis factor superfamily member 15), and CRH (corticotropin releasing hormone) in network #1 are closely associated with the activation of the ERK1/2 signaling pathway [69-72]. Additional cellular factors, including MGAT3 (mannosyl glucosaminyltransferase 3), HS6ST1 (heparin sulfate 6-O-sulfotransferase 1), and CIRBP (cold inducible RNA binding protein), that activate ERK 1/2 by increased phosphorylation are also upregulated in vaccine ILTV infection [73-75]. These results indicate that the mitogenic signaling pathway may be activated by increased phosphorylation of ERK 1/2 in vaccine ILTV infection, resulting in weak cytopathic effects that facilitate the recovery of cellular proliferation capabilities of embryonic lung cells during vaccine ILTV infection. In addition, several enzymatic antioxidants in lung cells that would reduce cellular oxidative stress included SOD3 (superoxide dismutase 3, extracellular), GPX7 (glutathione peroxidase 7), and GSTs (glutathione S tranferases; GST-A3, -T1, -A4) were also assigned to network #1. This up-regulation of these enzymatic antioxidants would serve to protect cells from oxidant-mediated cell death. These results concur with a previous report of enhanced endogenous antioxidant protection during vaccine ILTV infection and attenuation of cell death [76]. Focus molecules found in network #1 can be summarized by the following functions: stimulation of various secreting proteins, inhibition of the cell death pathway, and attenuation of oxidative stress.

Functional interpretation of other networks is described in Additional files 3 and 4.

### Comparison of host responses of virulent strain and vaccine ILTV infection

**Figure 5:**
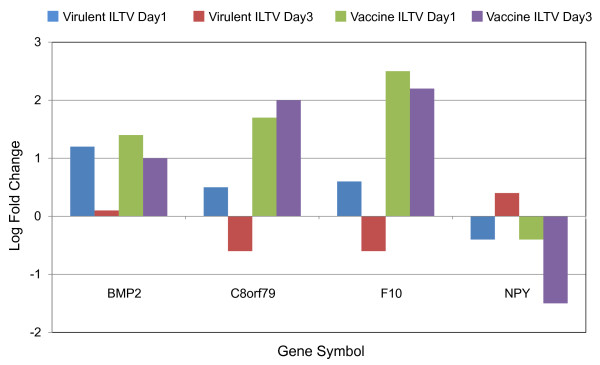
**Four differently expressed genes in both virulent strain and vaccine ILTV infections**. Relative expression was compared for four genes between vaccine ILTV infection and that reported for virulent strain ILTV [25]. The blue, red, green and purple bars represent virulent strain at 1 dpi, virulent strain at 3 dpi, vaccine at 1 dpi, and vaccine at 3 dpi for ILTV infections, respectively.

Previously, we reported 273 differentially expressed chicken genes mapped by the IPA program for virulent ILTV infection and the analysis of the functions and molecular networks of these genes [25]. To find host cellular mechanisms common to both virulent strain and vaccine ILTV infection, the 213 differentially expressed genes that responded to vaccine ILTV infection in the present study were compared to the 273 differentially expressed genes found in the previous virulent ILTV infection study. The results showed that 25 genes overlapped in both virulent strain and vaccine ILTV infections. Of these 25 genes, 21 showed a similar differential expression pattern as found in the virulent ILTV infection at 1 and 3 dpi (the GenBank accession, gene symbol and gene name are listed in Additional file 5). Interestingly, 4 of 25 genes were regulated in the distinct direction for expression patterns at 1 or 3 dpi between virulent and vaccine ILTV in the present study (see Figure 5). Those genes included bone morphogenetic protein 2 (BMP2, AY237249), chromosome 8 open reading frame 79 (C8orf79, CR390951), coagulation factor × (F10, D00844), and neuropeptide Y (NPY, M87294).

Bone morphogenetic proteins (BMPs), as members of the transforming growth factor beta (TGFβ) superfamily, are involved in various cellular functions including cell type specification, differentiation, pluripotency, apoptosis, proliferation, and tissue morphogenesis [77]. Deregulation of BMP signaling is also implicated in disease states including cancer [78]. Though the functional role of BMP in ILTV infection is unknown, BMPs including BMP2 were found to mediate herpesviral reactivation found in the EBV positive cell line [79]. The expression and secretion of BMP2 was regulated by HIV proteins in differentiating mesenchymal stem cells [80]. Expression of BMP2 in both virulent and vaccine ILTV infections increased at 1dpi and the increased expression level in vaccine ILTV infection was maintained until 3dpi, while the expression in virulent ILTV was not changed at 3dpi (Figure 5). The continuous increase of BMP2 expression during ILTV vaccine infection may play a homeostatic role in maintaining cellular morphology against virus infection.

Neuropeptide Y (NPY) involves multiple cellular mechanisms related to both virus entry into the central nervous system (CNS) and virus-induced neurological diseases. NPY protects the nervous system from murine retrovirus-induced neurological disease [81]. The up-regulation of NPY was observed in both infection and reactivation of VZV from a latent infection in human sensory trigeminal and dorsal ganglia, which are both sensory neurons [82]. It suggests that the up-regulation of NPY in virulent ILTV infection at 3 dpi may facilitate the latent infection in the nervous system. In contrast, the continuous down-regulation of NPY in vaccine ILTV infection may delay the latent infection to protect the host from diseases.

The coagulation factor, F10, is highly expressed during the early phase of SARS (severe acute respiratory syndrome) corona virus infection in human peripheral blood mononuclear cells (PBMCs) [83]. Expression of F10 in vaccine ILTV infections of lung cells increased at 1- and 3dpi, but the expression in virulent ILTV was not changed significantly at either 1- or 3dpi (Figure 5). A functional role of increased expression and secretion of F10 in vaccine herpesvirus infected lung cells has not been studied to date. The C8orf79 is an ORF region on chicken chromosome 8, but its function is unknown.

## Conclusions

In this study, we examined genome-wide host cellular transcriptomic changes by vaccine ILTV infection. Unlike our previous research on virulent ILTV infection, the vaccine ILTV infection showed weak cytopathic effects, cellular morphology recovery after initial cytopathic effects were observed and very little cell death. Possible molecular interpretations confirmed the suppression of cell death and weak cytopathic effect production during vaccine ILTV infection. Four host cellular genes were significantly modulated by vaccine ILTV infection, and could represent important secreting regulators to protect cells from cytotoxic damages. Although host gene expression by vaccine ILTV infection was determined in a cell culture system and not by an *in vivo *vaccination study, the results in this study provide important insights into differential host cellular defense mechanisms by regulating host gene expression response of vaccine ILTV infection to compare those of virulent ILTV infection.

## Methods

### Cell culture and vaccine ILTV infection

Primary chicken embryo lung cells were prepared as previously described [25]. All cell culture reagents were purchased from Invitrogen Life Technologies (Carlsbad, CA). Cells were maintained at 37°C in a 5% CO_2 _incubator in 10 cm culture dishes by passaging every 3-4 days in 10 ml growth medium consisting Dulbecco's Modified Eagle Medium (DMEM, 0.45% glucose) plus 10% fetal bovine serum (FBS), 100 units/ml penicillin, 100 μg/ml streptomycin, and 2 mM L-glutamine. A modified live ILTV commercial vaccine, LT-Blen (Merial Limited, Duluth, GA), was used to infect the chicken embryonic lung cells by the amount equal to 3 vaccination doses. After incubation of infected cells for 1 h with gentle rocking every 15 min, growth medium was added to each culture dish. The cells were incubated for up to 4 days. The protocols used in this study were approved by both the Institutional Biosafety Committee (IBC; permit number: 10007) of the University of Arkansas and the Animal and Plant Health Inspection Service (APHIS; permit number: 102743) of the United States Department of Agriculture (USDA).

### Total RNA extraction

TRIzol reagent (Invitrogen Life Technologies, Carlsbad, CA) was used to extract total RNA from uninfected- or vaccine ILTV infected chicken embryonic lung cells from 1 to 4 days post infection (dpi). Total RNA treated by DNase I (New England BioLabs Inc., Ipswich, MA) was re-purified by TRIzol reagent and quantified using a Nanodrop1000 spectrophotometer (Thermo Scientific, Wilmington, DE) and qualified quality assessed by agarose gel (data not shown). To validate vaccine ILTV infection, expression of UL35 and US5 genes, in addition to chicken GAPDH (a host gene expression control), were determined by end-point reverse transcription PCR with gene specific primers. PCR amplicons were analyzed by agarose gel electrophoresis and images were obtained using the GelDoc system (Biorad, Hercules, CA).

### Probe labeling and microarray hybridization

Initially, 2 μg of total RNA was used to synthesize Cy3 or Cy5 labeled complementary RNA (cRNA) using the Two Color Microarray Quick Labeling kit (Agilent Technologies, Palo Alto, CA) following the manufacturer's instructions and as described previously [25]. To avoid possible dye effects, RNA Spike-in controls, which were synthesized from the Adenovirus E1A transcriptomes containing different concentrations of dye in each set [33], were added to RNA samples as Spike-in A for Cy3 and Spike-in B for Cy5 and mixed with un-infected control and vaccine ILTV infected samples at each dpi, respectively. For the additional control of dye effects, the dyes were swapped in two of four total replicates to confirm further hidden dye effects. Each 825 ng of Cy3 and Cy5 labeled cRNA probes were co-hybridized on a 4X44K Agilent chicken oligo microarray (array ID: 015068). After washing and drying, the slides were scanned using a Genepix 4000B scanner (Molecular Devices Corporation, Sunnyvale, CA) with the tolerance of saturation at 0.005%.

### Microarray data analysis

Background-corrected red and green intensities for each spot were used in subsequent analyses. Global normalization based on locally weighted linear regression (LOWESS) was applied to the intensities by removing effects which arise from undesirable systematic variations in microarray experiments. The ratio of signal intensities of corresponding spots to all Spike-ins used were compared to reference ratios reported previously [33]. All normalized data were deposited in the Gene Expression Omnibus (GEO; accession number: GSE30269). Genes showing both signal to noise ratio (SNR) of > 3 (meaning foreground signals are three times greater than background signals), and foreground intensity of > 100 at all time points were considered as reliable signals. To identify differentially expressed genes throughout the four dpi, normalized fold change values were subjected to statistical analysis by a one-way ANOVA test in JMP Genomics 5.0 http://www.jmp.com/software/genomics/ licensed to the Cell and Molecular Biology (CEMB) program of the University of Arkansas. Low fold change values, which were less than two fold differences in all four dpi, were not considered as differential expression. Fold change values representing differential expression were displayed as log_2 _conversions.

### Quantitative reverse transcription-polymerase Chain reaction (qPCR)

To verify the microarray data, qPCR was performed with 18 genes using gene specific primer sets designed by Primer3 software http://frodo.wi.mit.edu/cgi-bin/primer3/primer3.cgi, which were synthesized by Integrated DNA Technologies (Coralville, IA). Primer information is shown in Table 1. Three μg of total RNA, which was used for the microarray analysis, was converted to cDNA and qPCR was performed under the following conditions: 40 cycles of denaturing 95°C for 30 s, annealing at 58 - 62°C for 1 min, extending at 72°C for 30 s, and finally extending at 72°C for 10 min. A non-template control (NTC) and an endogenous loading control (chicken GAPDH) were used for the relative quantification. The fold change values for the vaccine ILTV infected groups compared to uninfected control were determined by the -ΔΔCT method, which is comparable to log_2 _values in microarray [84].

### Bioinformatics

The Ingenuity Pathways Analysis (IPA) software version 9.0 (Ingenuity Systems^®^; http://www.ingenuity.com) was used to study biological functions and molecular interactions among differentially expressed genes. IPA analyzes various bioinformatics tools including functional annotation, clustering, and network discovery based on Ingenuity Knowledge Base, which is the core technology of all IPA systems and the p-value developed from Right-tailed Fisher's exact test were mainly considered to interpret the interaction and functions of the differentially expressed genes [85,86]. The network analysis was limited to 10 networks and 35 molecules in each network to concentrate on the closest interacting focus molecules (focus genes = a subset of uploaded significant genes having direct interactions with other genes in the database) within the differentially expressed genes [87].

## Competing interests

The authors declare that they have no competing interests.

## Authors' contributions

JYL designed and performed experiments, analyzed the data, and wrote the manuscript. WB contributed the bioinformatics analysis using the IPA program and manuscript editing. BWK supervised all processes of the research, initial data analysis and manuscript editing. All authors read and approved the final manuscript.

## Supplementary Material

Additional file 1 **All 933 differentially expressed genes at four dpi time points of vaccine ILTV infection**. The Agilent ID, GenBank accession, gene symbol, log_2 _fold change (FC), mean log_2 _FC of four dpi, and their standard deviations (std) are listed. Click here for file

Additional file 2 **All 213 differentially expressed genes collected by IPA program**. The gene expression levels at all time points were determined by log_2 _fold change. All gene symbols and gene names were generated based on the IPA database and confirmed with the Unigene function of NCBI. Click here for file

Additional file 3 **GenBank accessions, functions, and major focus molecules in four molecular networks**. (A) The top functions of each network, scores and the number of focus molecules are listed in each network. (B) GenBank accession numbers and gene symbols used in network analysis were listed for each network. Focus molecules were bolded and GenBank accession number were provided for focus molecules only. (C) The potentially important focus molecules in each network are listed. Click here for file

Additional file 4 **Four gene networks**. Symbols of functions for each molecules used to generate molecular networks are displayed. (A) network #1 (B) network #2 (C) network #3 (D) network #4. Enlarged images for each dpi are attached. The green indicates down-regulation, while the red depicts up-regulation. Different color intensities represent levels of log_2 _fold change in the designated molecule. Click here for file

Additional file 5 **The 21 genes showing a similar expression pattern in both virulent strain and vaccine ILTV infection**. Click here for file
