# Supplementary material for: Genome-wide host responses against infectious laryngotracheitis virus vaccine infection in chicken embryo lung cells
Source: BMC Genomics. 2012 Apr 24;13:143. doi: 10.1186/1471-2164-13-143 (PMC3353197; doi:10.1186/1471-2164-13-143)
Supplement: Additional file 3 — GenBank accessions, functions, and major focus molecules in four molecular networks. (A) The top functions of each network, scores and the number of focus molecules are listed in each network. (B) GenBank accession numbers and gene symbols used in network analysis were listed for each network. Focus molecules were bolded and GenBank accession number were provided for focus molecules only. (C) The potentially important focus molecules in each network are listed. [file 1471-2164-13-143-S3.DOC]

**Additional file 3. Top functions and focus molecules in four molecular networks**.

**A. The top functions of each network**

| ID | Top Functions | Score | # Focus Molecules |
| --- | --- | --- | --- |
| 1 | Free Radical Scavenging, Lipid Metabolism, Small Molecule Biochemistry | 44 | 25 |
| 2 | Inflammatory Response, Post-Translational Modification, Tissue Development | 32 | 20 |
| 3 | Cellular Movement, Hematological System Development and Function, Immune Cell Trafficking | 26 | 17 |
| 4 | Post-Translational Modification, Protein Folding, Cell Death | 21 | 17 |

**B. GenBank accessions of focus molecules in four molecular networks**

| **Network 1** | | **Network 2** | |
| --- | --- | --- | --- |
| **GenBank** | **Symbol** | **GenBank** | **Symbol** |
| **CR406458** | **AMY2A** | **M94271** | **AGRN** |
|  | Amylase |  | Alp |
| **AJ719361** | **ARHGAP15** | **AB082935** | **ANKRD1** |
| **AJ251273** | **CCK** | **AJ829443** | **AQP5** |
| **DQ126684** | **CIRBP** |  | BMP |
| **AJ621492** | **CRH** | **AY237249** | **BMP2** |
| **BX930381** | **EMP1** | **DQ097308** | **BMP3** |
|  | ERK1/2 |  | C1q |
| **AF479650** | **FIGF** |  | Collagen Alpha1 |
|  | FXR ligand |  | Collagen type I |
| **AB105812** | **GEM** |  | Collagen type IV |
| **BX936196** | **GJB1** |  | CYP17 |
|  | Glutathione peroxidase |  | Elastase |
|  | glutathione transferase | **BX934260** | **EPHX2** |
| **BX933973** | **GPX7** | **AJ851612** | **ERAP1** |
| **L15386** | **GSTA3** |  | Fcer1 |
| **AF133251** | **Gsta4** | **U34977** | **FMOD** |
| **U13676** | **GSTT1** | **X87609** | **FST** |
|  | hCG | **Y09235** | **GLRX** |
| **AB093515** | **HS6ST1** | **CR733243** | **GREM1** |
|  | Lh |  | Iga |
| **Z94720** | **LSAMP** | **U26946** | **INHBA** |
| **AJ851531** | **MGAT3** |  | Laminin |
| **CR390466** | **NMU** | **M80584** | **LUM** |
| **AB033829** | **PDGFC** | **AF022226** | **MBL2** |
| **CR390309** | **PLTP** | **AJ719326** | **MMP7** |
| **M61145** | **PRNP** | **AJ719310** | **NCOA7** |
| **AF151967** | **RGS20** |  | NFkB (complex) |
|  | SAA |  | Notch |
| **AJ720139** | **SDF4** | **CR353565** | **POSTN** |
|  | Sod |  | Smad |
| **BX930609** | **SOD3** | **BX950598** | **TFPI** |
| **BX930081** | **TNFSF15** |  | Tgf beta |
|  | VLDL | **NM_001031045** | **TGFB2** |
| **X80207** | **VLDLR** | **CR352482** | **TNFRSF11B** |

| **Network 3** | | **Network 4** | |
| --- | --- | --- | --- |
| **GenBank** | **Symbol** | **GenBank** | **Symbol** |
| **AJ851685** | **ADAM28** |  | 26s Proteasome |
|  | Akt | **CR733321** | **ABCC2** |
|  | Alpha catenin | **Z11961** | **BCL2** |
|  | Calpain |  | Caspase |
| **AB032767** | **CD9** | **BX935188** | **CCDC109B** |
| **BX931737** | **COL19A1** | **AF019142** | **CYP24A1** |
| **CR733397** | **COL4A3** | **CR524127** | **DNAJA1** |
| **CR385318** | **COL5A2** | **BX936068** | **DNAJC19** |
| **X15041** | **COL6A2** |  | ERK |
| **CR387830** | **COL8A1** | **AY723747** | **FKBP5** |
|  | collagen |  | Histone h4 |
| **BX950717** | **EGFL6** | **AJ720813** | **HNRNPD** |
| **BX935456** | **EGLN3** |  | HSP |
|  | Estrogen Receptor |  | Hsp70 |
| **AF051399** | **FBLN1** |  | Hsp90 |
| **BX933997** | **FHL2** | **X70101** | **HSP90AB1** |
|  | Fibrinogen | **CR523558** | **HSPD1** |
| **U21327** | **FN1** | **AY591922** | **IGFBP1** |
|  | Hsp27 |  | Ikb |
|  | IFN alpha/beta |  | MAP2K1/2 |
|  | IL1 |  | Mek |
|  | Integrin |  | Mlc |
|  | Integrin alpha 3 beta 1 | **CR353595** | **MYL10** |
|  | Mmp | **X13862** | **MYL3** |
| **BX929570** | **MMP23B** |  | Nfat (family) |
|  | NFkB (family) | **BX950381** | **NR2C1** |
|  | P38 MAPK | **CR387492** | **PDK4** |
|  | Pro-inflammatory Cytokine |  | PP2A |
| **AJ719603** | **RAP1B** |  | Rar |
| **CR386571** | **SMURF2** |  | Rock |
| **J00902** | **SPINK5** |  | Rxr |
| **M60853** | **THBS2** | **AF003631** | **TYRP1** |
|  | Tlr |  | Ubiquitin |
|  | Tnf |  | VitaminD3-VDR-RXR |
|  | Trypsin | **Z47206** | **ZBTB16** |

**C. Functions of focused molecules in each network**

| ID | Related functions with main focus molecules |
| --- | --- |
| 1 | - Oxidative stress reduction genes including superoxide dismutase (SOD) 3 [1, 2] and glutathione peroxidase including GPX7 [3]. - Lipid metabolism including PLTP (phospholipid transfer protein) and VLDLR (very low density lipoprotein receptor). - Neuronal system protein, neuromedin U (NMU). |
| 2 | - NFκB related signaling mechanisms including AGRN (agrin) [4], ANKRD1 (ankyrin repeat domain-containing protein 1) [5],GLRK (glutathione protein-disulfide reductase) [6], NCOA7 (nuclear receptor coactivator 7) [7], ERAP1 (endoplasmic reticulum aminopeptidase 1) [8], and MBL2 (mannose-binding lectin 2) [9]. - TGF beta superfamily including TGFB2 (transforming growth factor beta 2), BMP2 [10], BMP3 [11], and associated factors including GREM1 (Gremlin 1) [12] and POSTN (periostin) [13]. |
| 3 | - Cell structural proteins to maintain cell morphology and cell membrane functions including fibronectin 1 (FN1) [14], fibulin 1(FBLN1) [15], collagens (COL-19A1, -4A3, -5A2, -6A2, -8A1) [16], and tissue factor pathway inhibitor (TFPI) [17]. |
| 4 | - Heat shock proteins including HSPAB1, DNAJA1 [DnaJ (Hsp40) homolog, member 1], DNAJC19 [DnaJ (Hsp40) homolog, member 19], and HSPD1 (heat shock 60kDa protein 1), as molecular chaperons, increasing their expression under stress conditions [18, 19] and a related protein including FK506 binding protein 5 (FKBP5) [20]. - Apoptosis and angiogenesis including BCL2 (B-cell lymphoma 2) [21]. - Insulin-like growth factor-binding protein 1 (IGFBP1) [22]. |

**References**

1. Zelko IN, Mariani TJ, Folz RJ: **Superoxide dismutase multigene family: a comparison of the CuZn-SOD (SOD1), Mn-SOD (SOD2), and EC-SOD (SOD3) gene structures, evolution, and expression.** *Free Radic.Biol.Med.* 2002, **33(3):**337-349.

2. Folz RJ, Crapo JD: **Extracellular superoxide dismutase (SOD3): tissue-specific expression, genomic characterization, and computer-assisted sequence analysis of the human EC SOD gene.** *Genomics* 1994, **22(1):**162-171.

3. Khan A, Tania M, Zhang D, Chen H: **Antioxidant enzymes and cancer.** *Chinese joural of Cancer Research* 2010, **22:**87-92.

4. Klein S, de Fougerolles AR, Blaikie P, Khan L, Pepe A, Green CD, Koteliansky V, Giancotti FG: **Alpha 5 beta 1 integrin activates an NF-kappa B-dependent program of gene expression important for angiogenesis and inflammation.** *Mol.Cell.Biol.* 2002, **22(16):**5912-5922.

5. Klein S, de Fougerolles AR, Blaikie P, Khan L, Pepe A, Green CD, Koteliansky V, Giancotti FG: **Alpha 5 beta 1 integrin activates an NF-kappa B-dependent program of gene expression important for angiogenesis and inflammation.** *Mol.Cell.Biol.* 2002, **22(16):**5912-5922.

6. Towne JE, Krane CM, Bachurski CJ, Menon AG: **Tumor necrosis factor-alpha inhibits aquaporin 5 expression in mouse lung epithelial cells.** *J.Biol.Chem.* 2001, **276(22):**18657-18664.

7. Minami T, Miura M, Aird WC, Kodama T: **Thrombin-induced autoinhibitory factor, Down syndrome critical region-1, attenuates NFAT-dependent vascular cell adhesion molecule-1 expression and inflammation in the endothelium.** *J.Biol.Chem.* 2006, **281(29):**20503-20520.

8. Forloni M, Albini S, Limongi MZ, Cifaldi L, Boldrini R, Nicotra MR, Giannini G, Natali PG, Giacomini P, Fruci D: **NF-kappaB, and not MYCN, regulates MHC class I and endoplasmic reticulum aminopeptidases in human neuroblastoma cells.** *Cancer Res.* 2010, **70(3):**916-924.

9. Elco CP, Guenther JM, Williams BR, Sen GC: **Analysis of genes induced by Sendai virus infection of mutant cell lines reveals essential roles of interferon regulatory factor 3, NF-kappaB, and interferon but not toll-like receptor 3.** *J.Virol.* 2005, **79(7):**3920-3929.

10. Chen D, Zhao M, Mundy GR: **Bone morphogenetic proteins.** *Growth Factors* 2004, **22(4):**233-241.

11. Bahamonde ME, Lyons KM: **BMP3: to be or not to be a BMP.** *J.Bone Joint Surg.Am.* 2001, **83-A Suppl 1(Pt 1):**S56-62.

12. Pereira RC, Economides AN, Canalis E: **Bone morphogenetic proteins induce gremlin, a protein that limits their activity in osteoblasts.** *Endocrinology* 2000, **141(12):**4558-4563.

13. Conway SJ, Doetschman T, Azhar M: **The inter-relationship of periostin, TGF beta, and BMP in heart valve development and valvular heart diseases.** *ScientificWorldJournal* 2011, **11:**1509-1524.

14. Grinnell F: **Fibronectin and wound healing.** *J.Cell.Biochem.* 1984, **26(2):**107-116.

15. Argraves WS, Tran H, Burgess WH, Dickerson K: **Fibulin is an extracellular matrix and plasma glycoprotein with repeated domain structure.** *J.Cell Biol.* 1990, **111(6 Pt 2):**3155-3164.

16. Muller WE, Schubert-Zsilavecz M: **Collagenin and collagen.** *Pharm.Unserer Zeit* 2004, **33(4):**267.

17. Broze GJ,Jr, Warren LA, Novotny WF, Higuchi DA, Girard JJ, Miletich JP: **The lipoprotein-associated coagulation inhibitor that inhibits the factor VII-tissue factor complex also inhibits factor Xa: insight into its possible mechanism of action.** *Blood* 1988, **71(2):**335-343.

18. Csermely P, Schnaider T, Soti C, Prohaszka Z, Nardai G: **The 90-kDa molecular chaperone family: structure, function, and clinical applications. A comprehensive review.** *Pharmacol.Ther.* 1998, **79(2):**129-168.

19. Chen XS, Zhang Y, Wang JS, Li XY, Cheng XK, Zhang Y, Wu NH, Shen YF: **Diverse effects of Stat1 on the regulation of hsp90alpha gene under heat shock.** *J.Cell.Biochem.* 2007, **102(4):**1059-1066.

20. Nair SC, Rimerman RA, Toran EJ, Chen S, Prapapanich V, Butts RN, Smith DF: **Molecular cloning of human FKBP51 and comparisons of immunophilin interactions with Hsp90 and progesterone receptor.** *Mol.Cell.Biol.* 1997, **17(2):**594-603.

21. Oltersdorf T, Elmore SW, Shoemaker AR, Armstrong RC, Augeri DJ, Belli BA, Bruncko M, Deckwerth TL, Dinges J, Hajduk PJ, Joseph MK, Kitada S, Korsmeyer SJ, Kunzer AR, Letai A, Li C, Mitten MJ, Nettesheim DG, Ng S, Nimmer PM, O'Connor JM, Oleksijew A, Petros AM, Reed JC, Shen W, Tahir SK, Thompson CB, Tomaselli KJ, Wang B, Wendt MD, Zhang H, Fesik SW, Rosenberg SH: **An inhibitor of Bcl-2 family proteins induces regression of solid tumours.** *Nature* 2005, **435(7042):**677-681.

22. Brinkman A, Groffen C, Kortleve DJ, Geurts van Kessel A, Drop SL: **Isolation and characterization of a cDNA encoding the low molecular weight insulin-like growth factor binding protein (IBP-1).** *EMBO J.* 1988, **7(8):**2417-2423.
