# Supplementary material for: Genome-wide host responses against infectious laryngotracheitis virus vaccine infection in chicken embryo lung cells
Source: BMC Genomics. 2012 Apr 24;13:143. doi: 10.1186/1471-2164-13-143 (PMC3353197; doi:10.1186/1471-2164-13-143)
Supplement: Additional file 4 — Four gene networks. Symbols of functions for each molecules used to generate molecular networks are displayed. (A) network #1 (B) network #2 (C) network #3 (D) network #4. Enlarged images for each dpi are attached. The green indicates down-regulation, while the red depicts up-regulation. Different color intensities represent levels of log2 fold change in the designated molecule. [file 1471-2164-13-143-S4.PPT]

## Slide 1
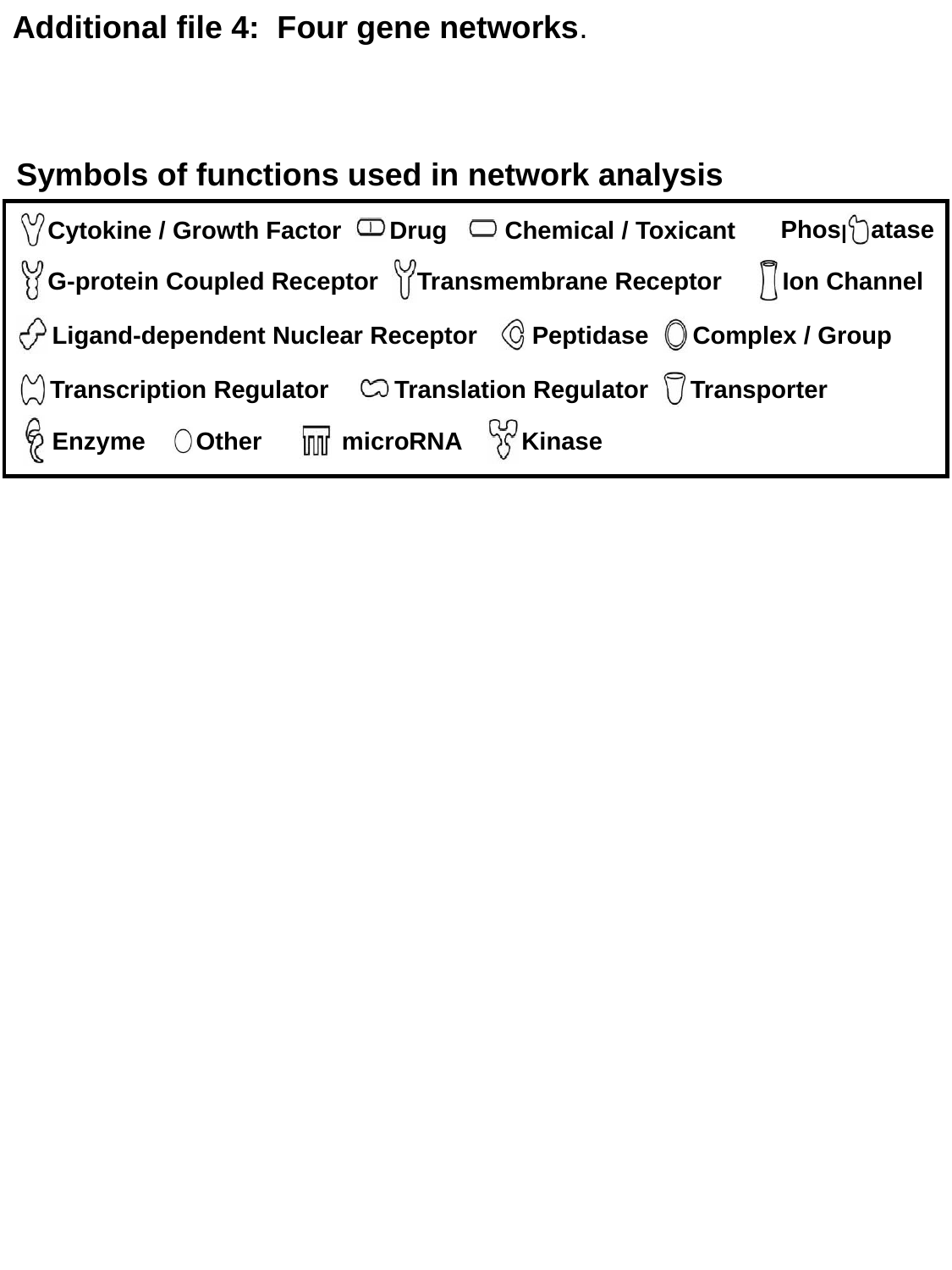

Additional file 4: Four gene networks.
Symbols of functions used in network analysis
Phosphatase
Cytokine / Growth Factor
Drug
Chemical / Toxicant
G-protein Coupled Receptor
Transmembrane Receptor
Ion Channel
Ligand-dependent Nuclear Receptor
Peptidase
Complex / Group
Transcription Regulator
Translation Regulator
Transporter
Enzyme
Other
microRNA
Kinase

## Slide 2
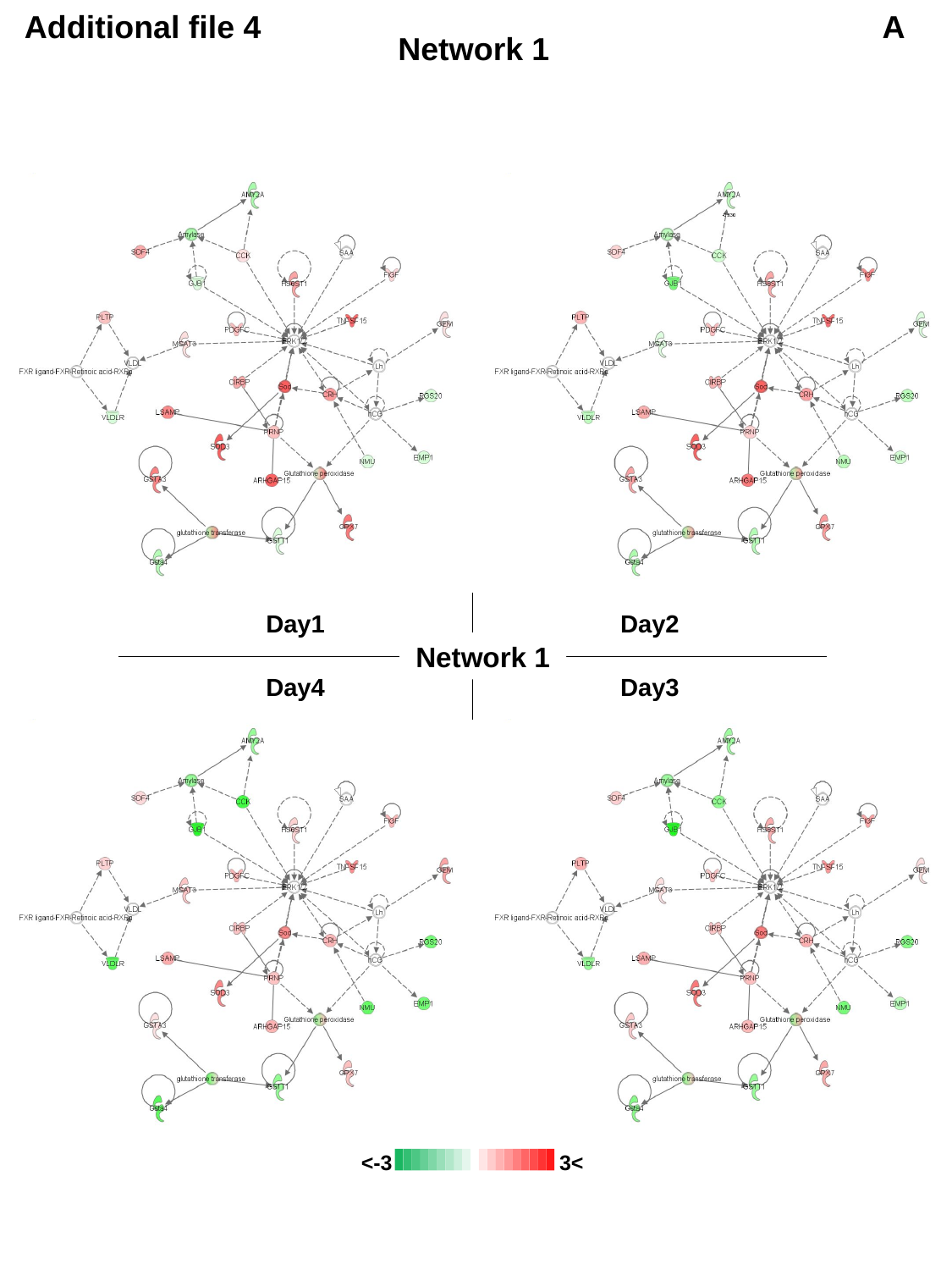

Additional file 4
A
Network 1
| Day1 | Day2 |
| --- | --- |
| Day4 | Day3 |
Network 1
<-3
3<

## Slide 3
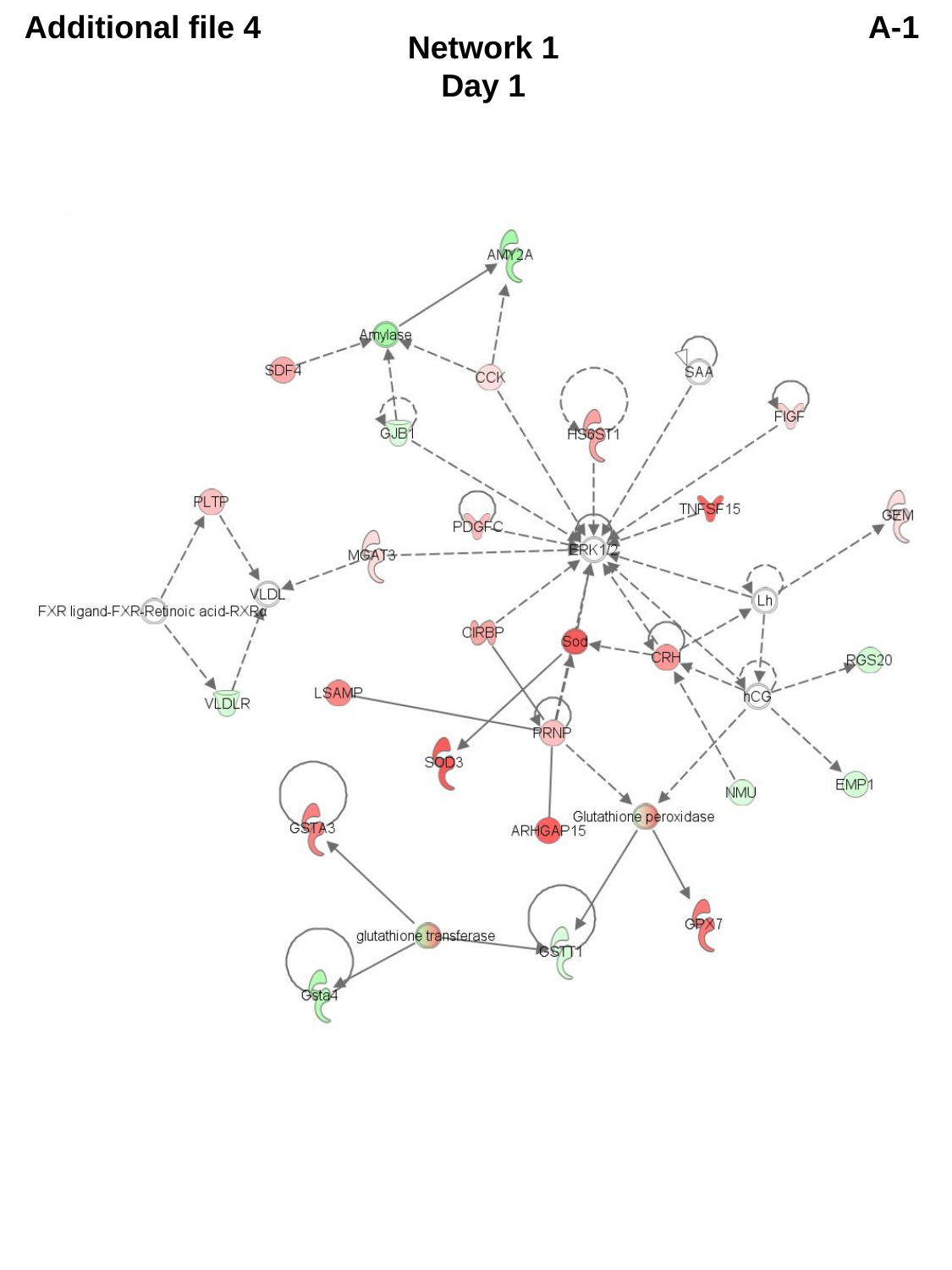

Additional file 4
A-1
Network 1
Day 1

## Slide 4
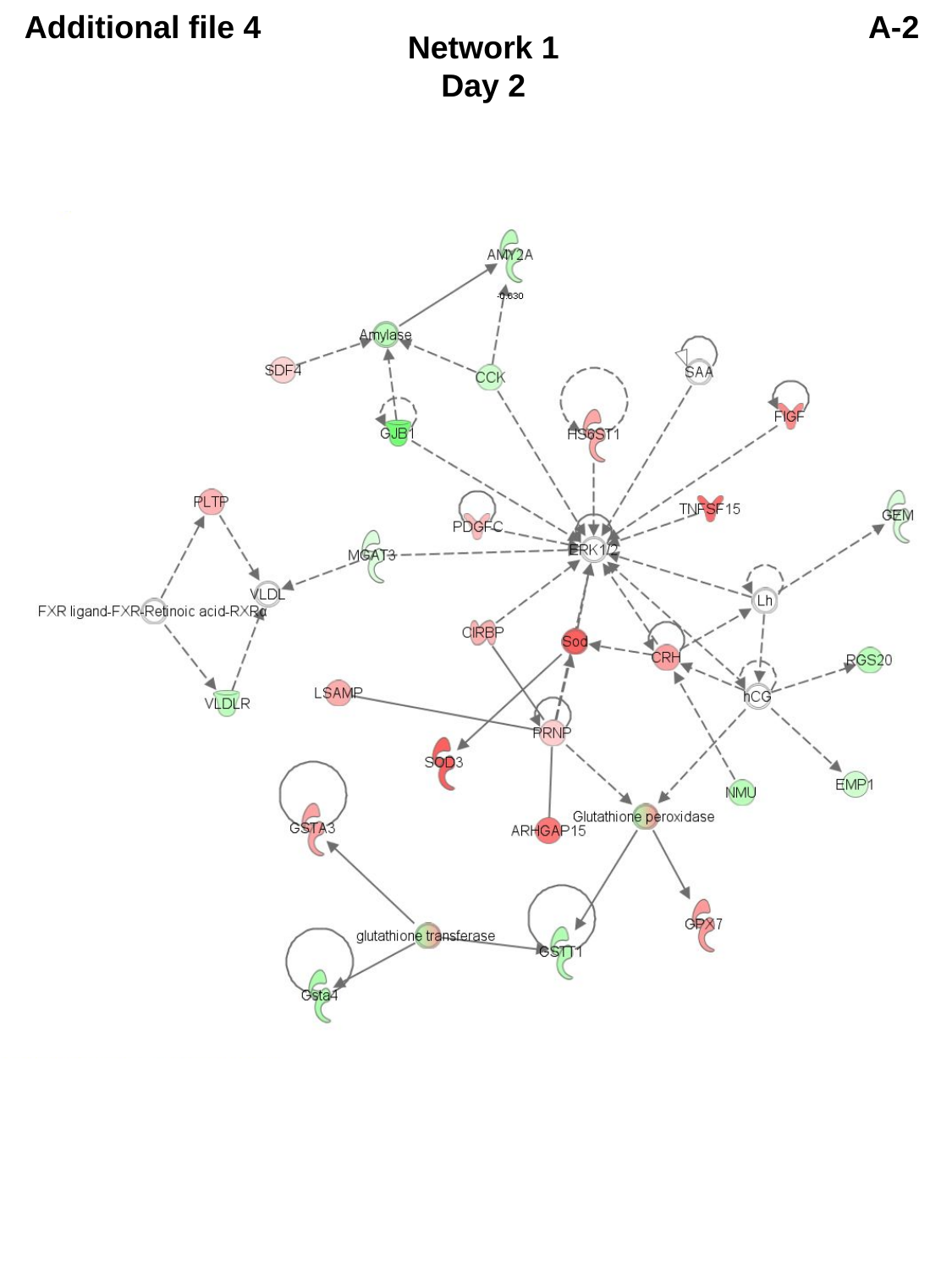

Additional file 4
A-2
Network 1
Day 2

## Slide 5
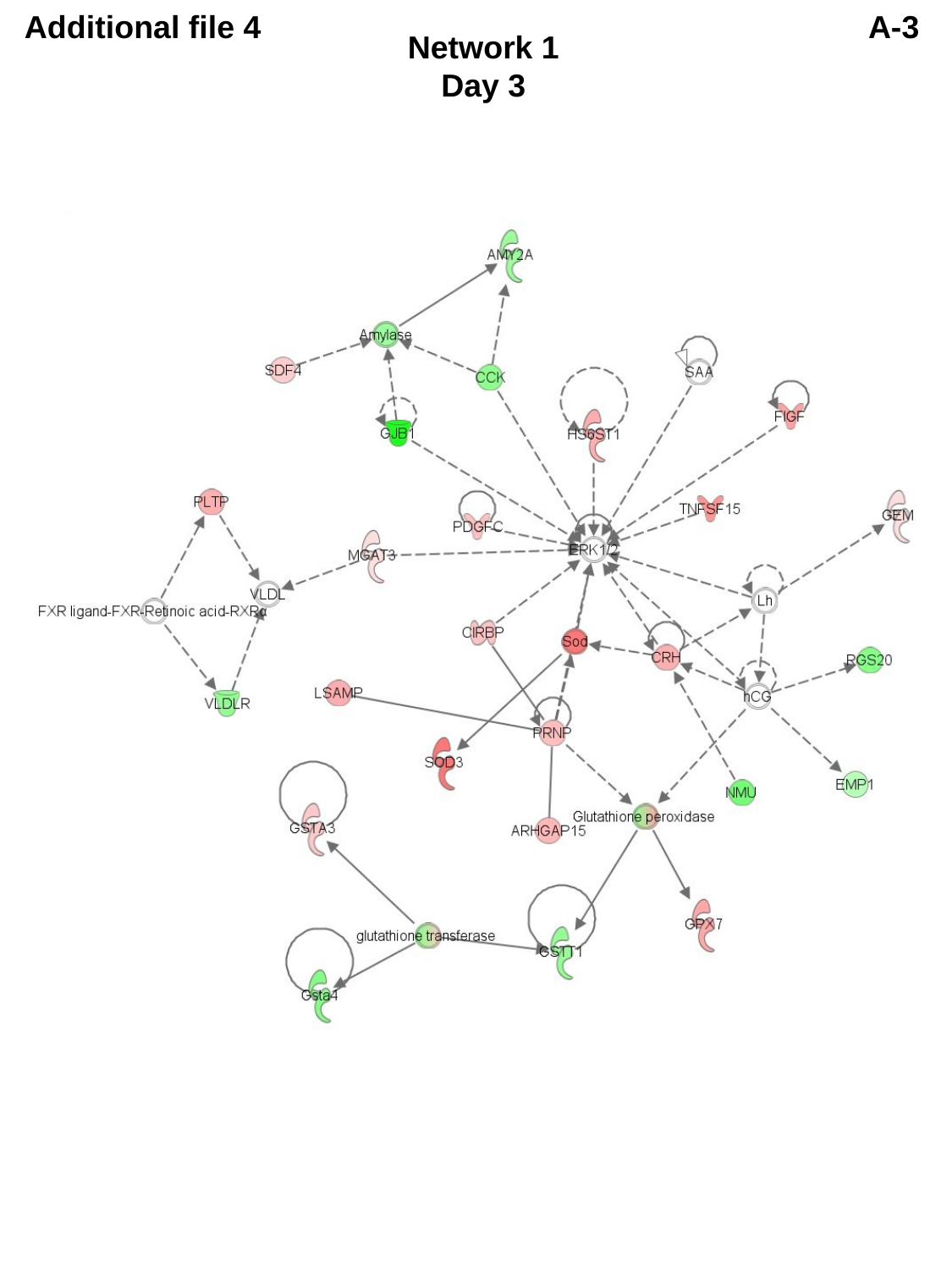

Additional file 4
A-3
Network 1
Day 3

## Slide 6
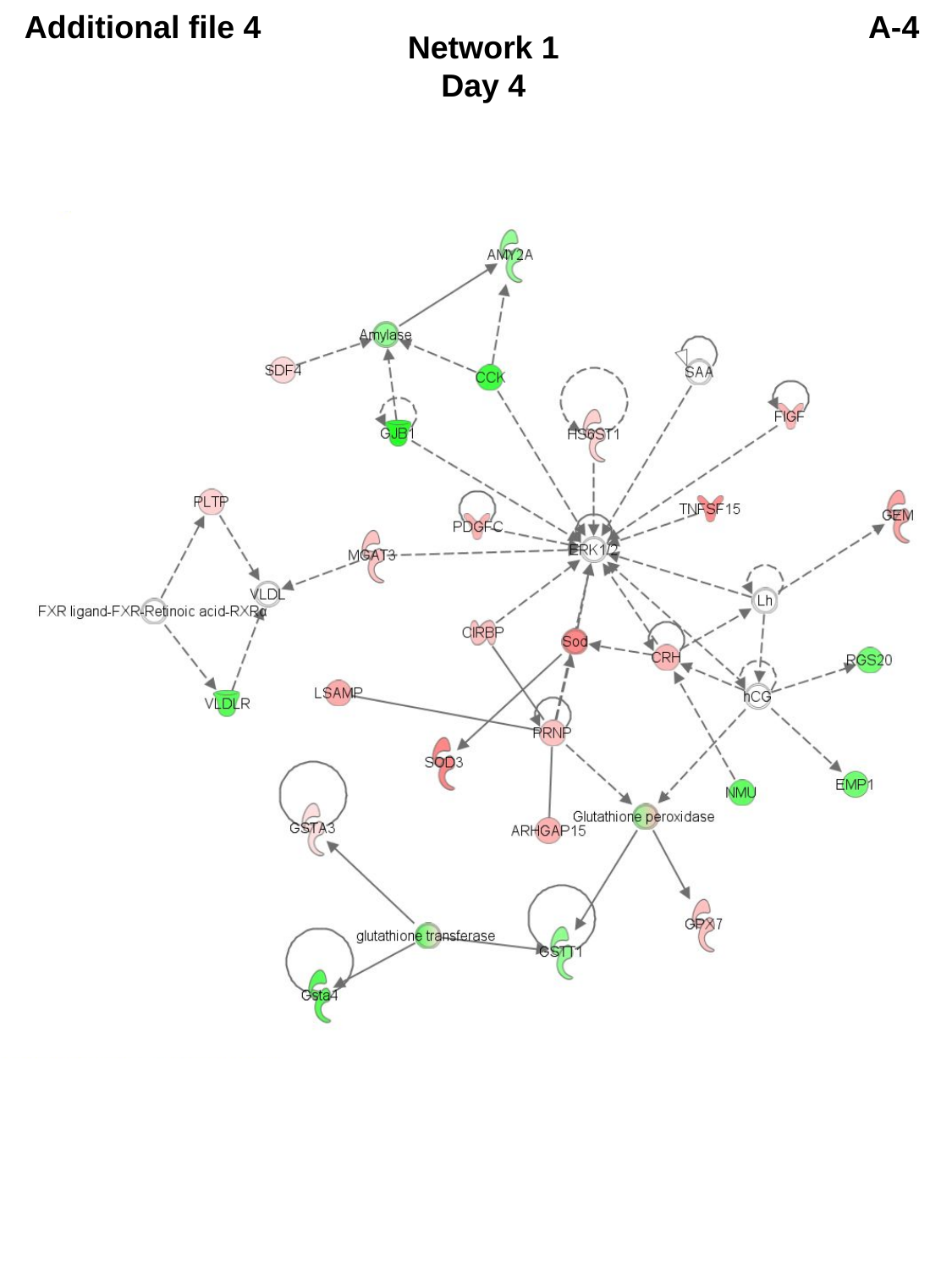

Additional file 4
A-4
Network 1
Day 4

## Slide 7
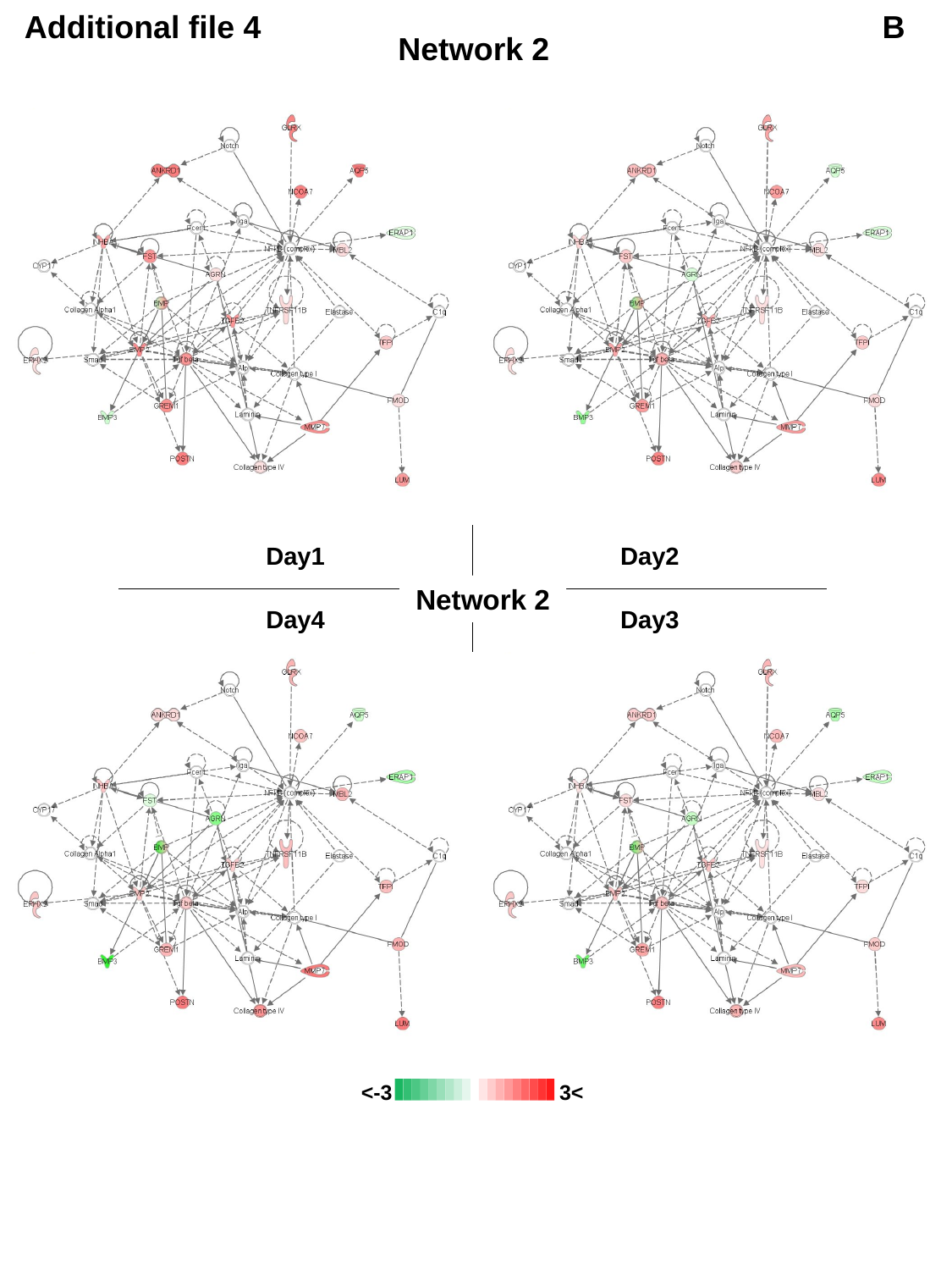

Additional file 4
B
Network 2
| Day1 | Day2 |
| --- | --- |
| Day4 | Day3 |
Network 2
<-3
3<

## Slide 8
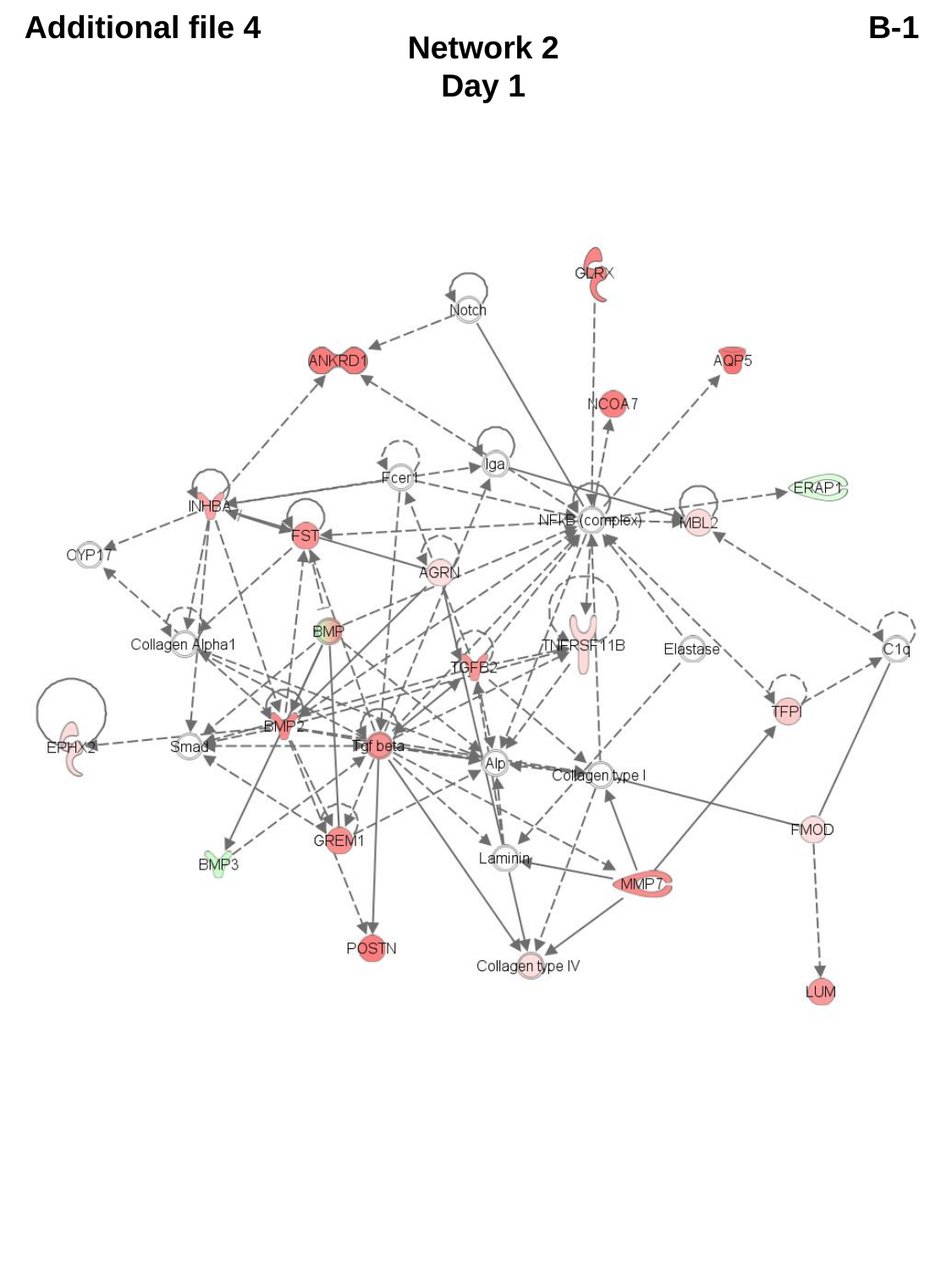

Additional file 4
B-1
Network 2
Day 1

## Slide 9
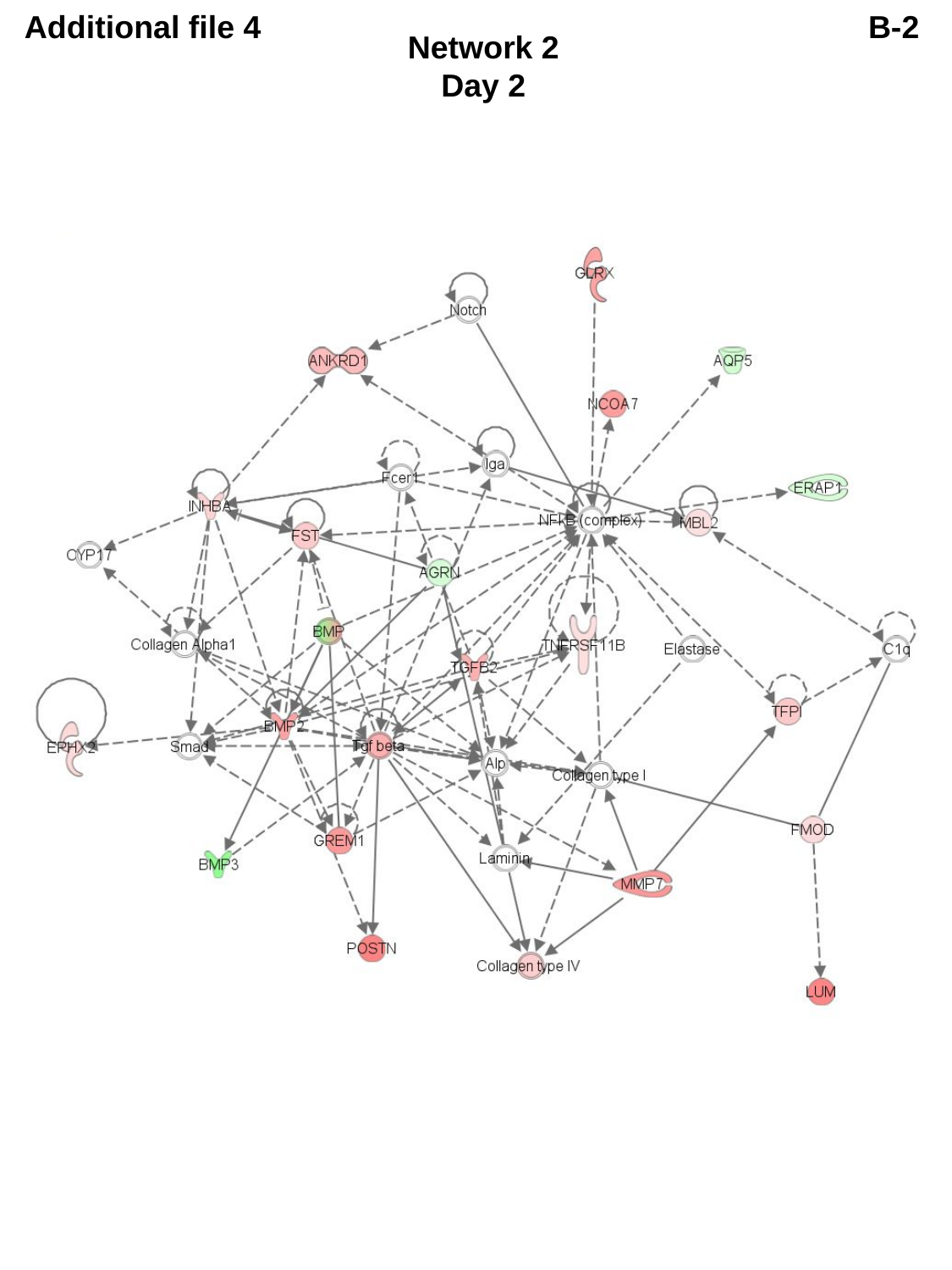

Additional file 4
B-2
Network 2
Day 2

## Slide 10
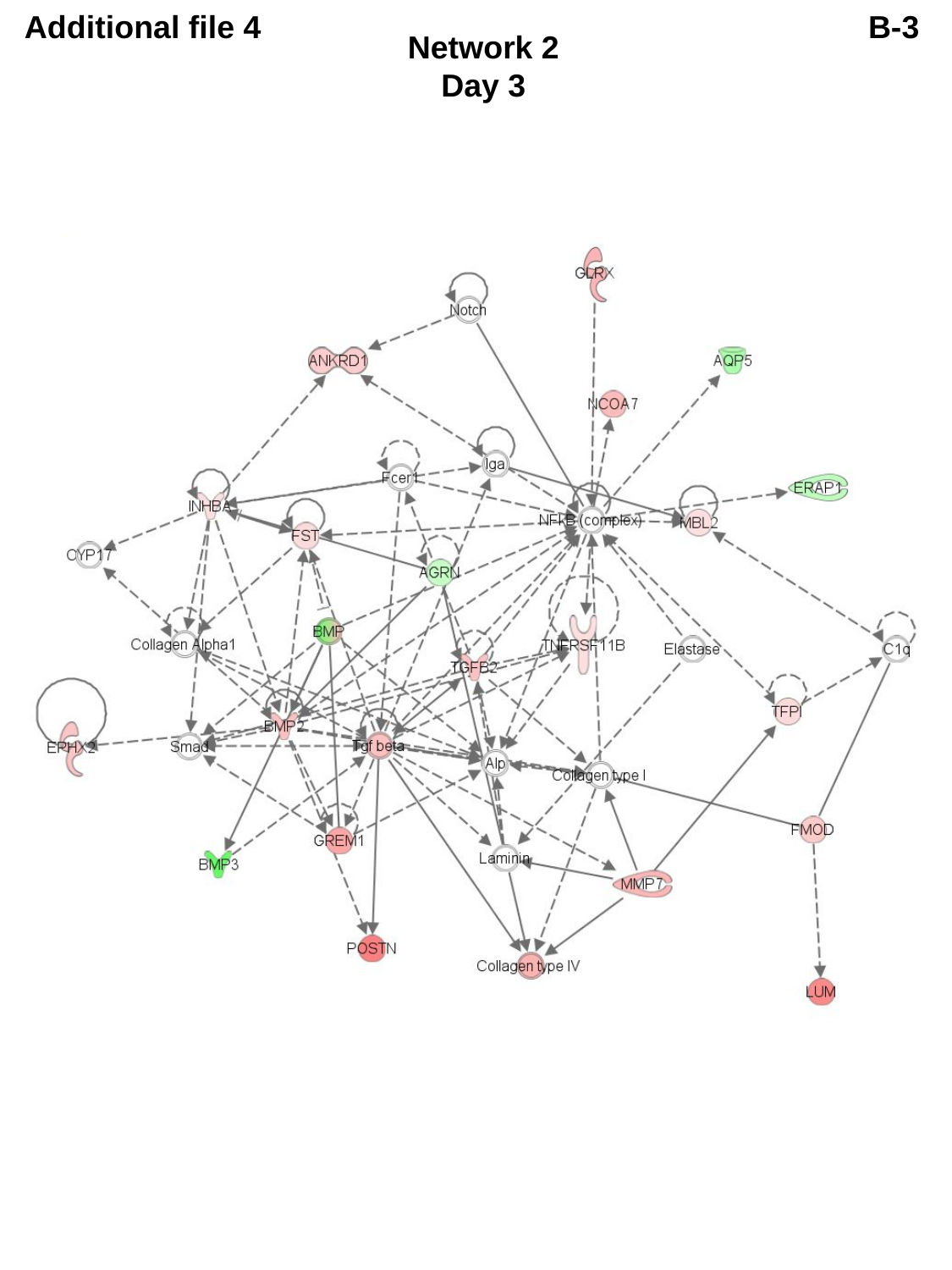

Additional file 4
B-3
Network 2
Day 3

## Slide 11
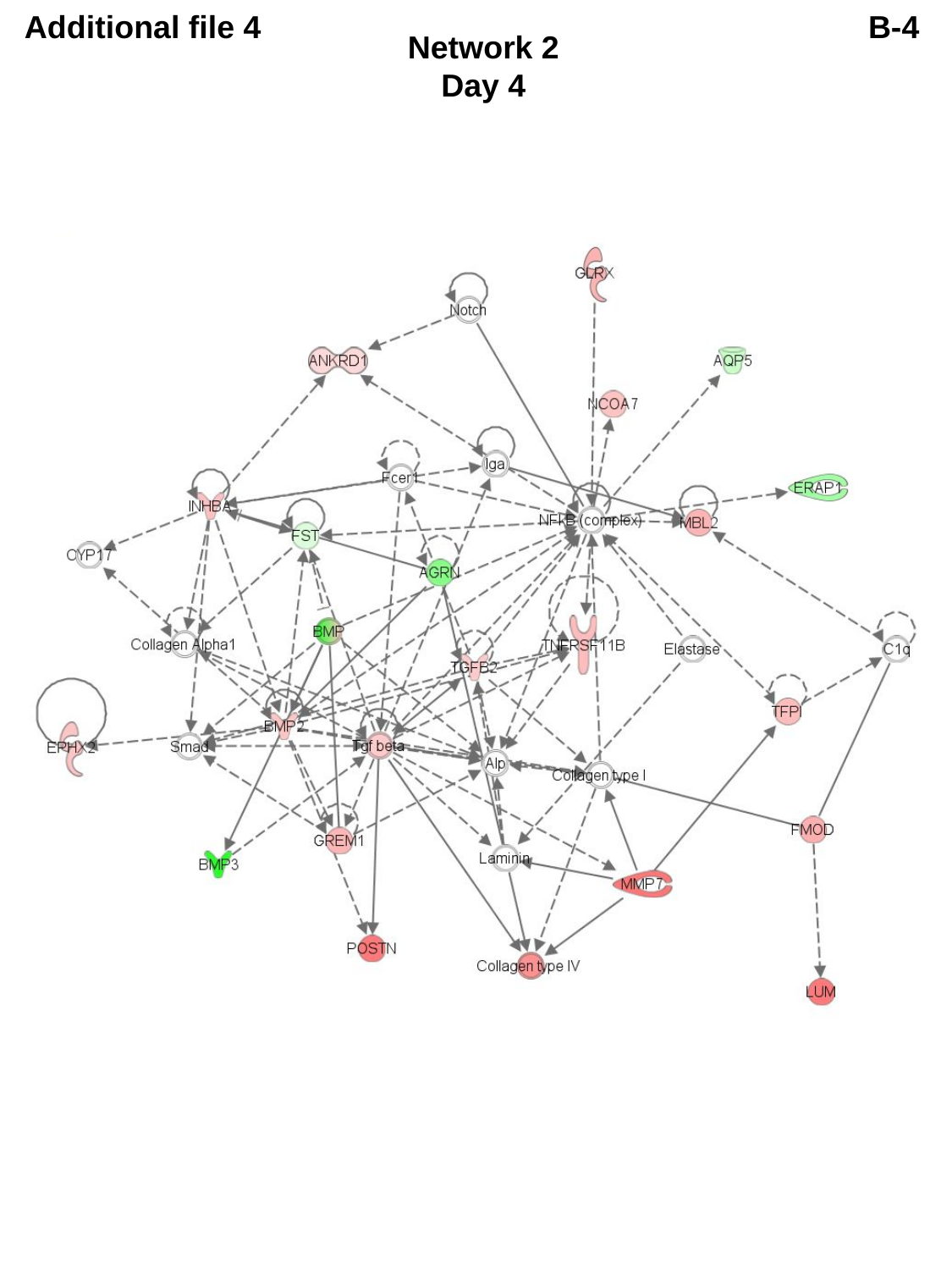

Additional file 4
B-4
Network 2
Day 4

## Slide 12
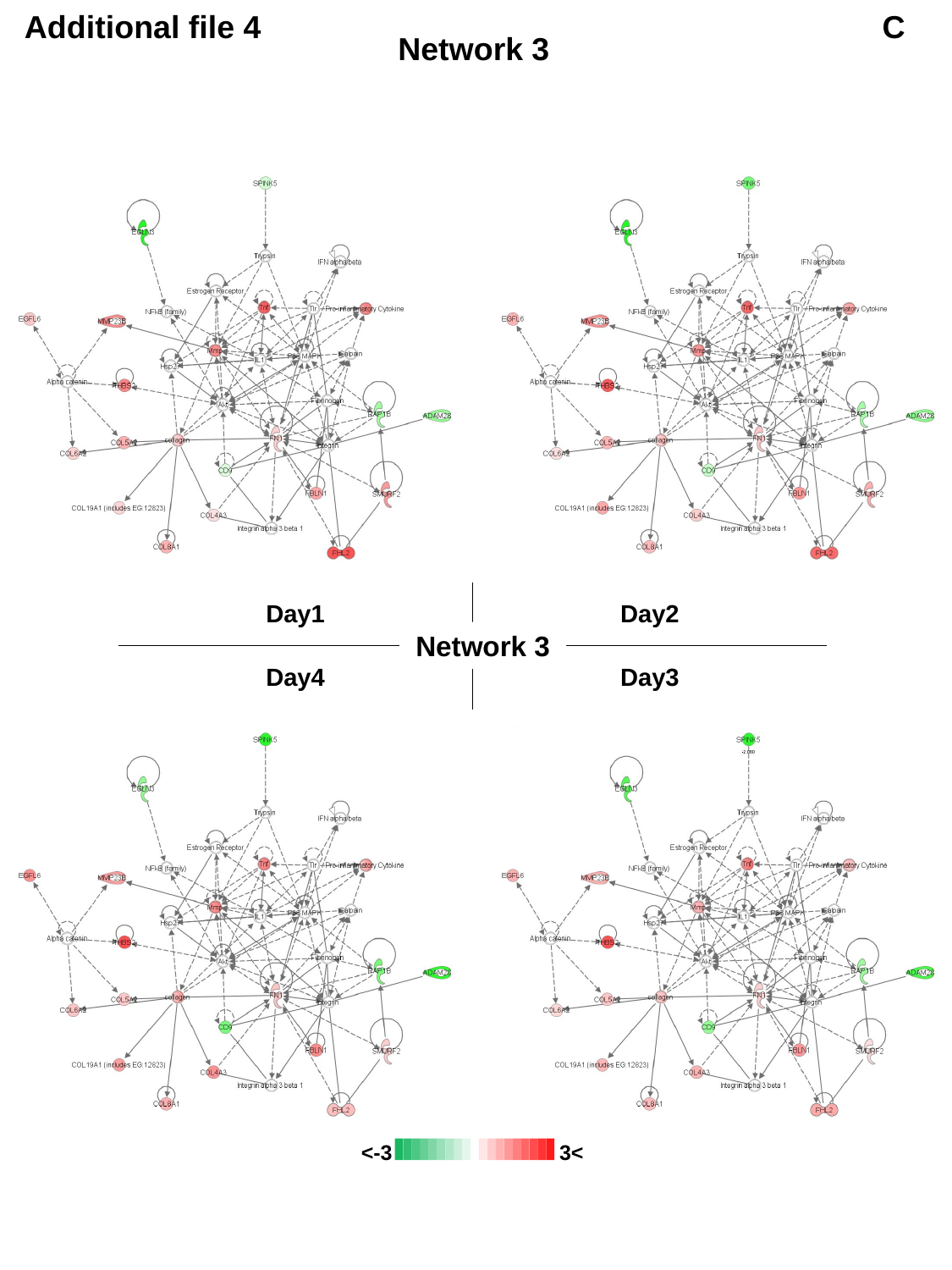

Additional file 4
C
Network 3
| Day1 | Day2 |
| --- | --- |
| Day4 | Day3 |
Network 3
<-3
3<

## Slide 13
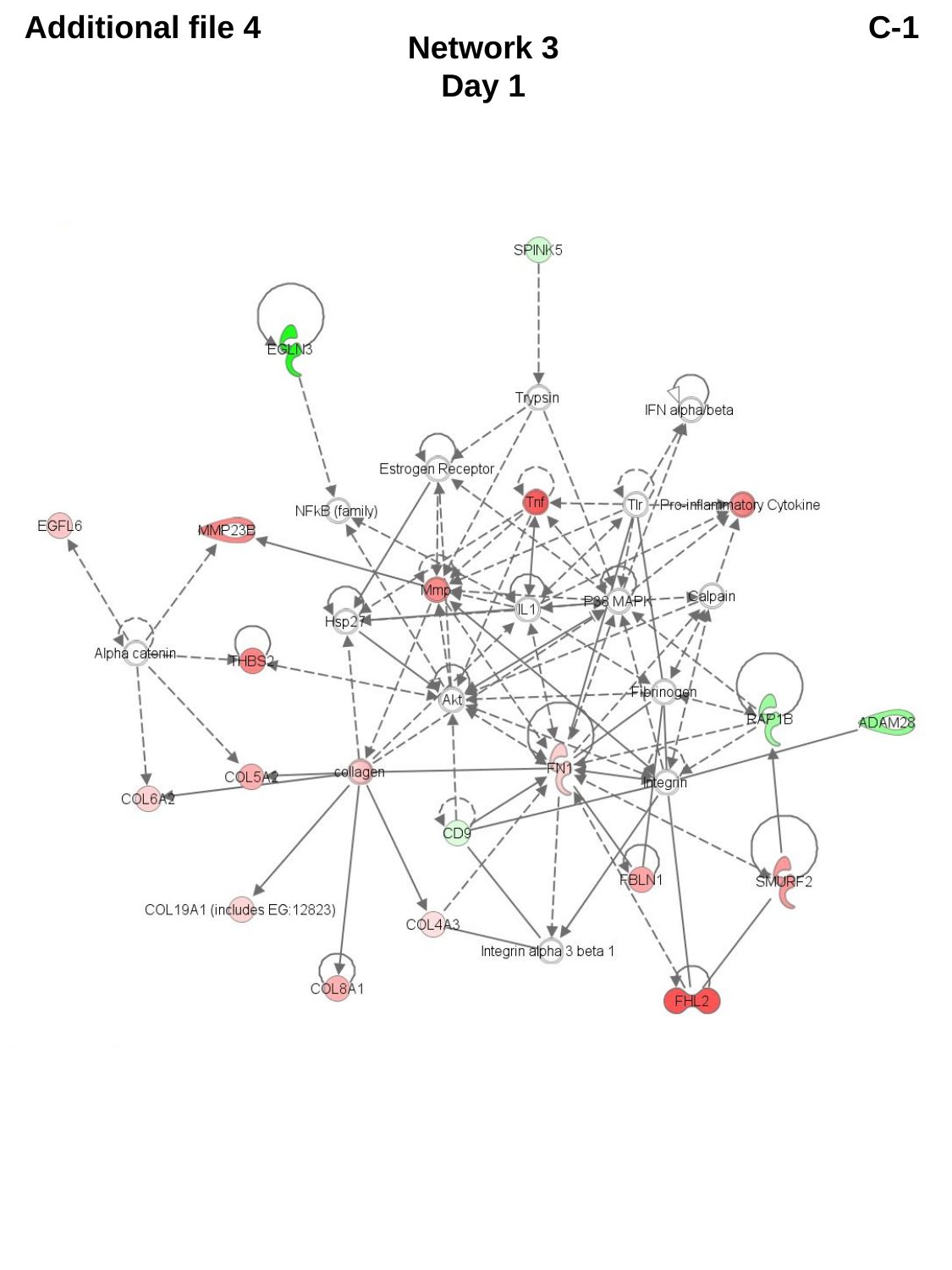

Additional file 4
C-1
Network 3
Day 1

## Slide 14
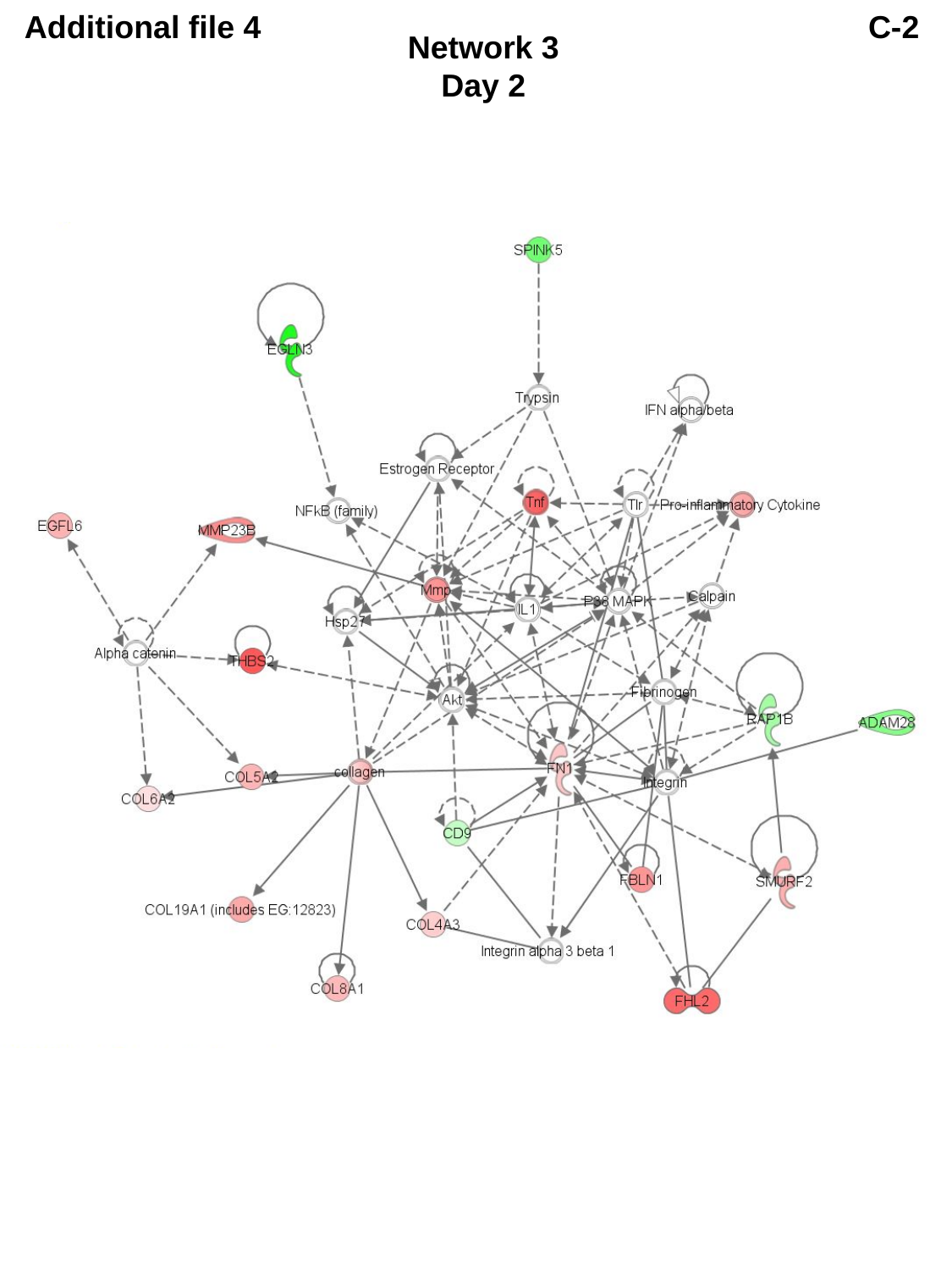

Additional file 4
C-2
Network 3
Day 2

## Slide 15
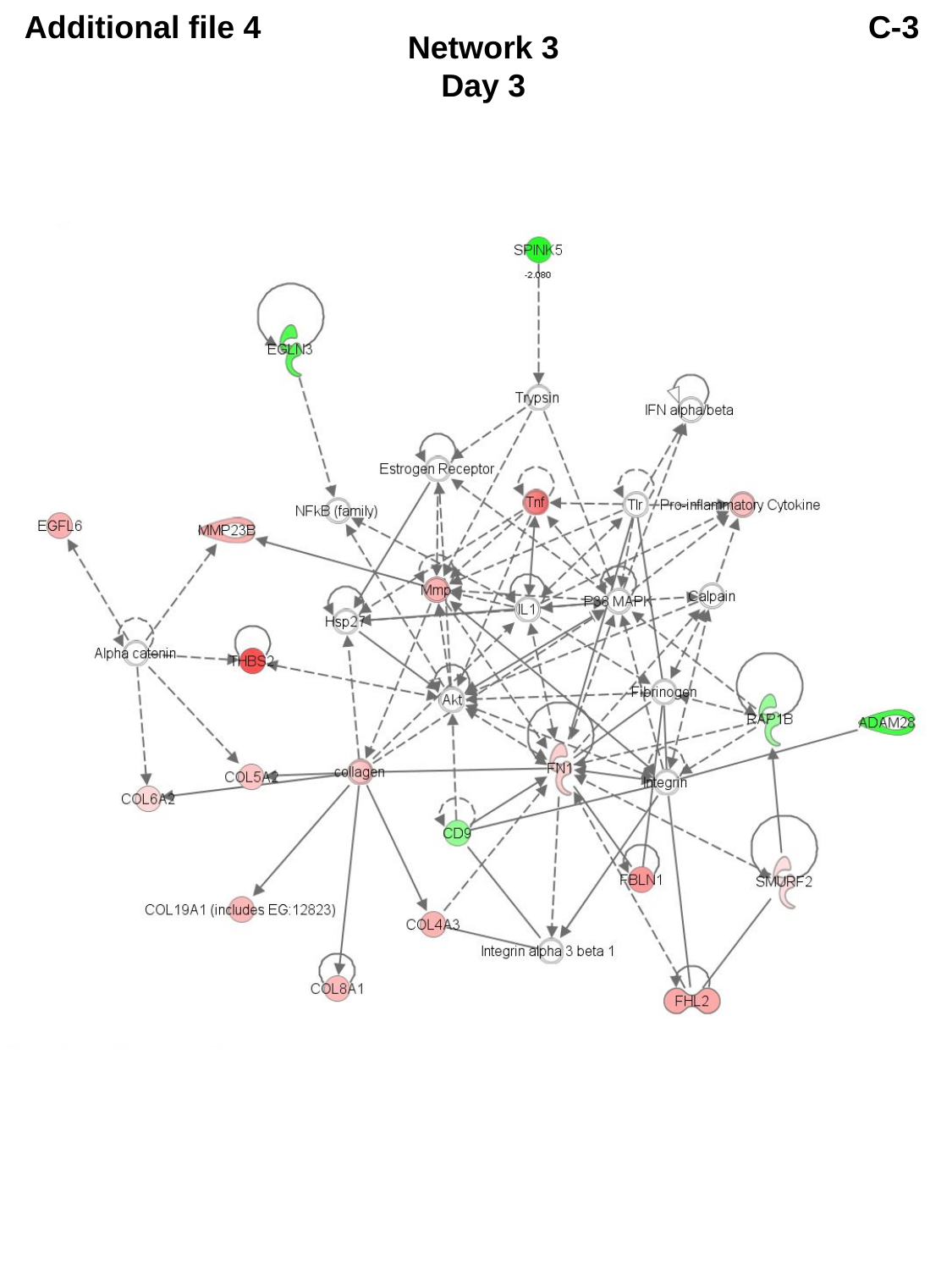

Additional file 4
C-3
Network 3
Day 3

## Slide 16
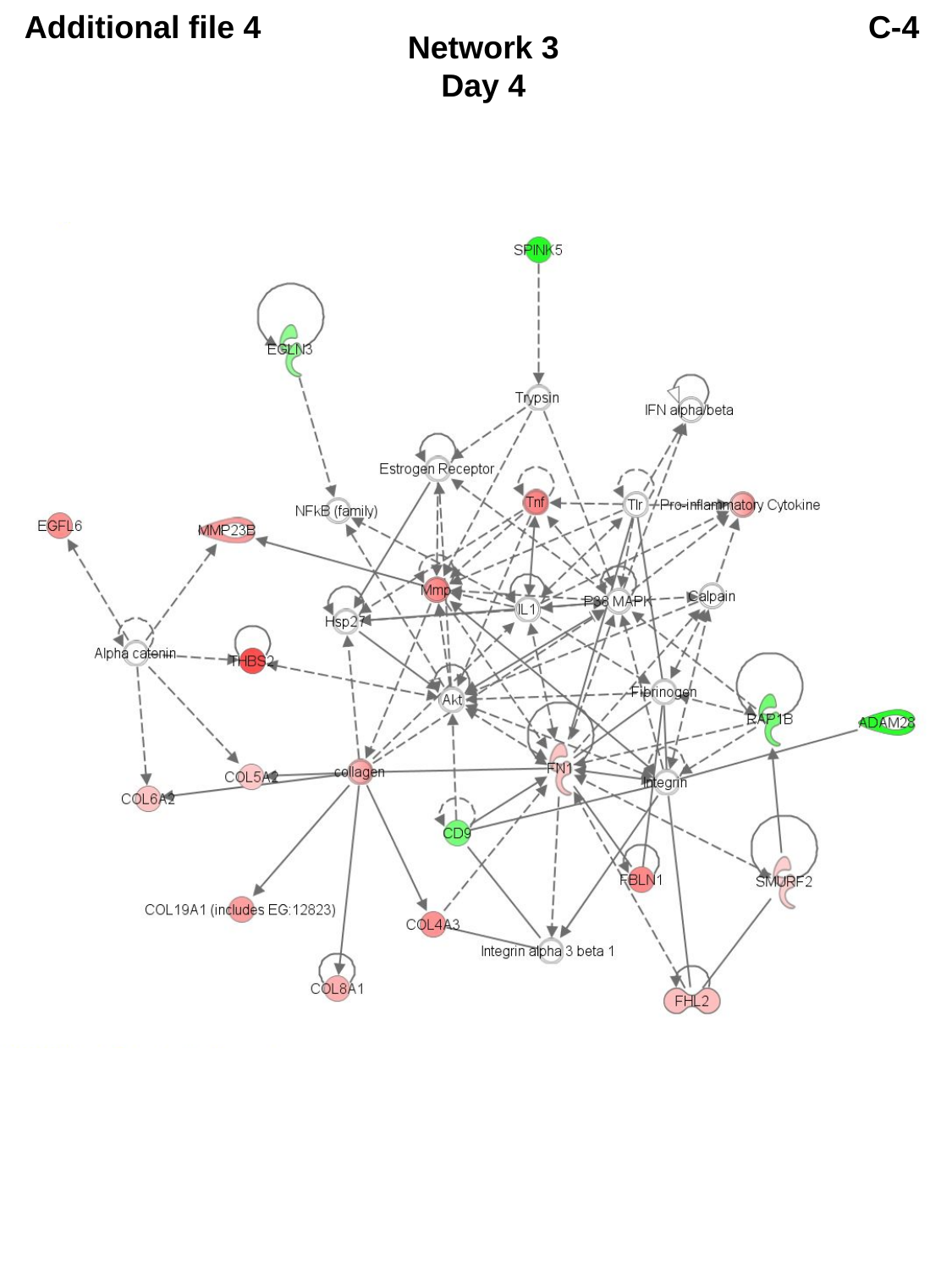

Additional file 4
C-4
Network 3
Day 4

## Slide 17
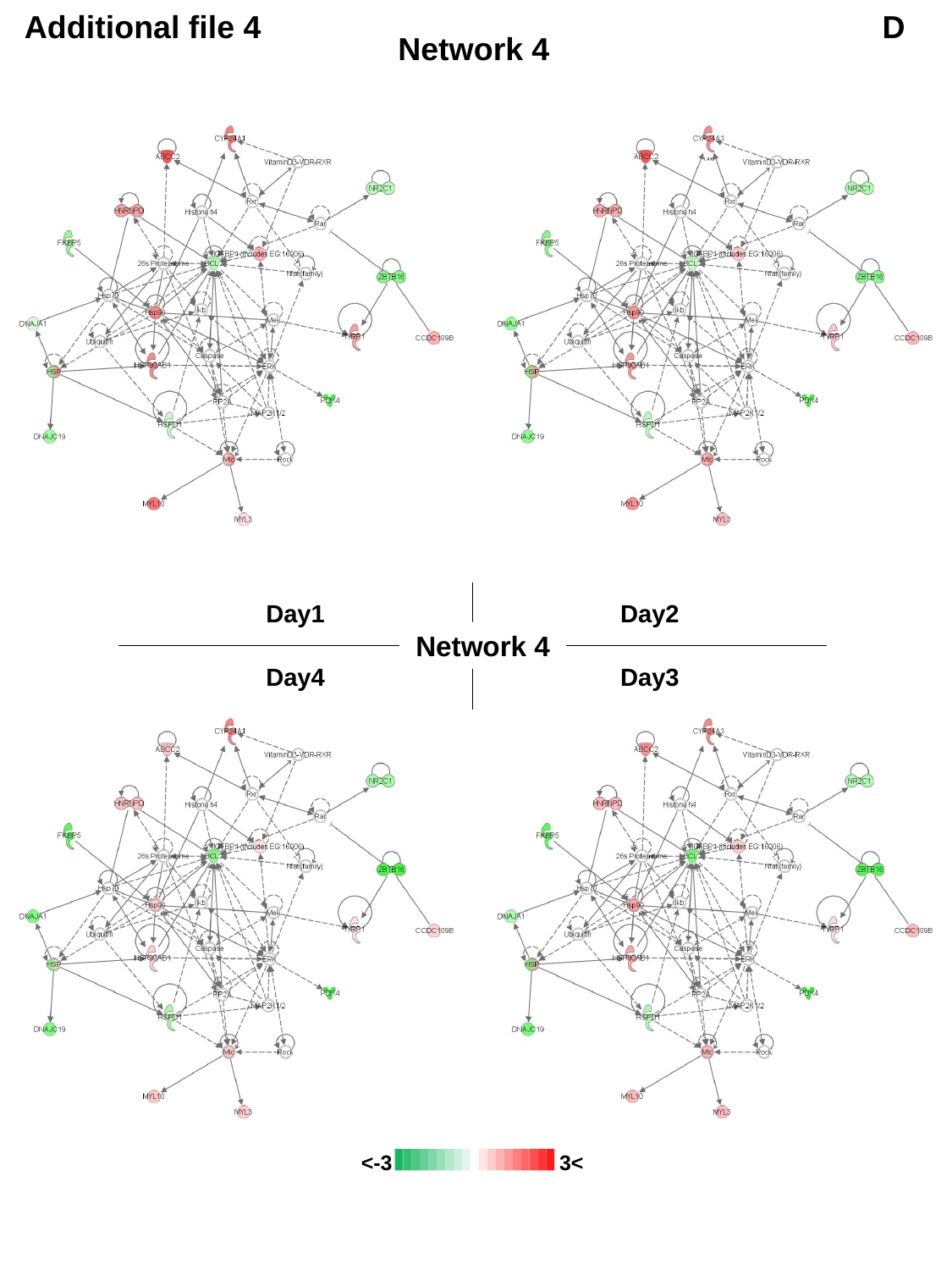

Additional file 4
D
Network 4
| Day1 | Day2 |
| --- | --- |
| Day4 | Day3 |
Network 4
<-3
3<

## Slide 18
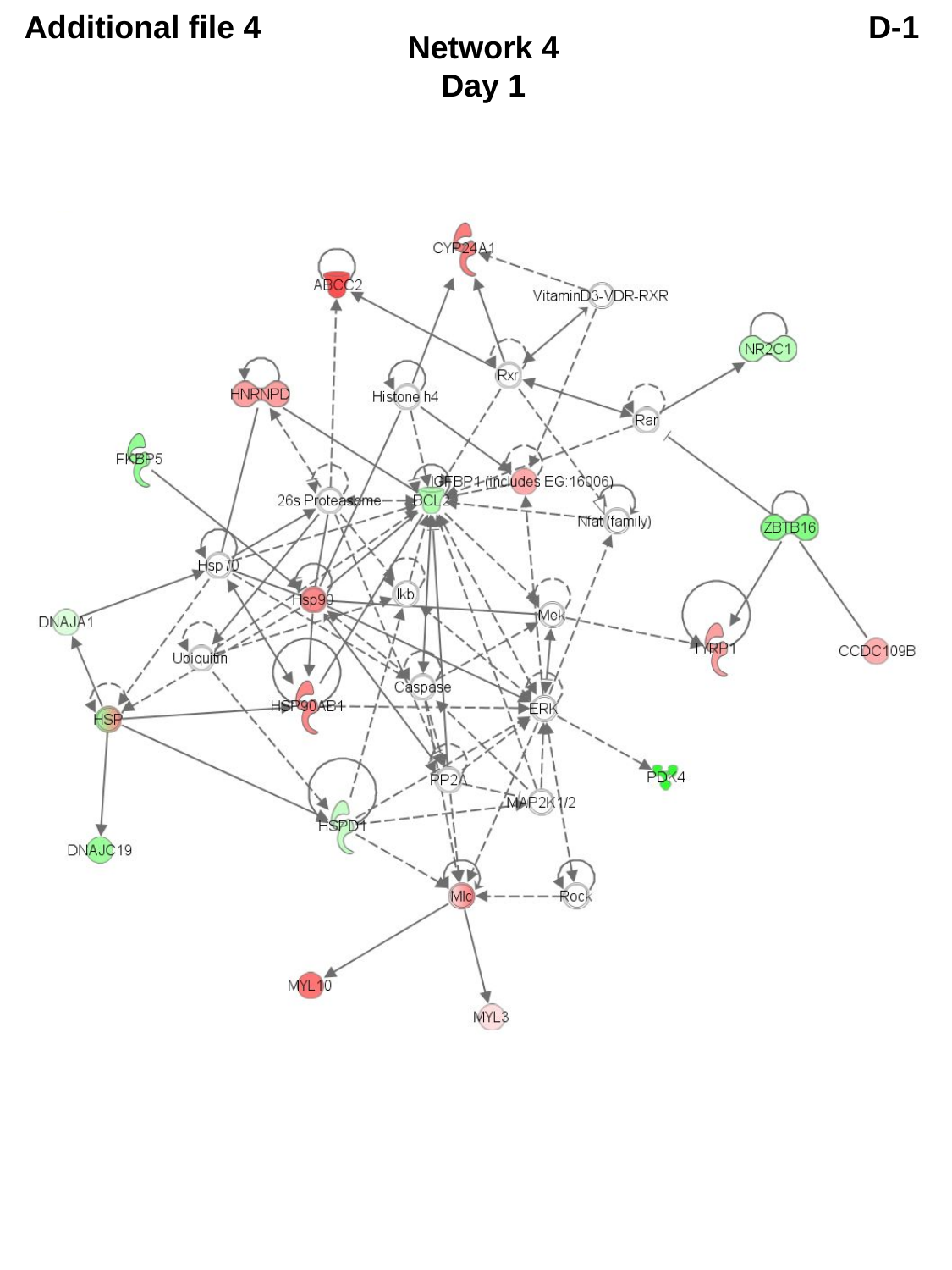

Additional file 4
D-1
Network 4
Day 1

## Slide 19
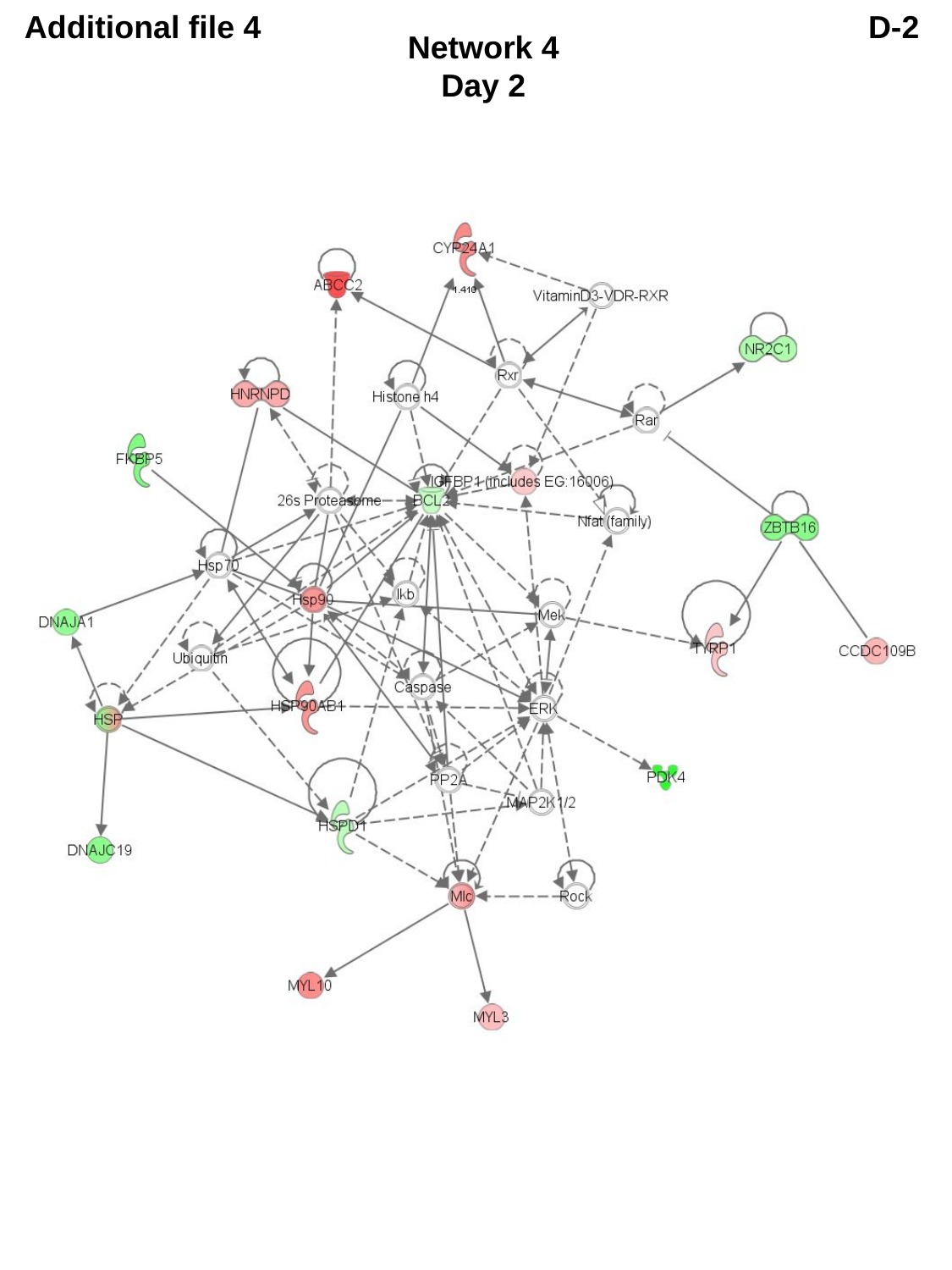

Additional file 4
D-2
Network 4
Day 2

## Slide 20
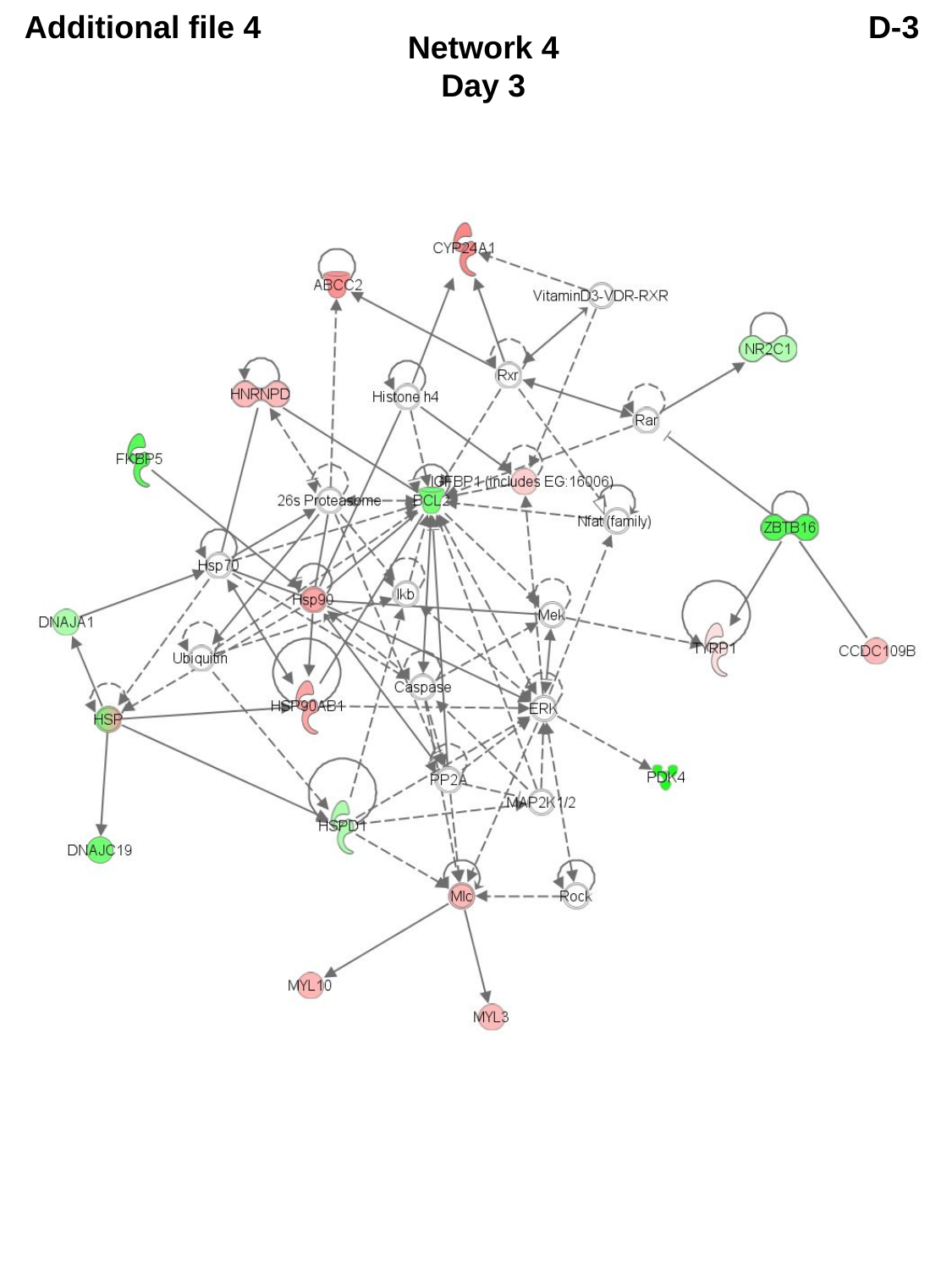

Additional file 4
D-3
Network 4
Day 3

## Slide 21
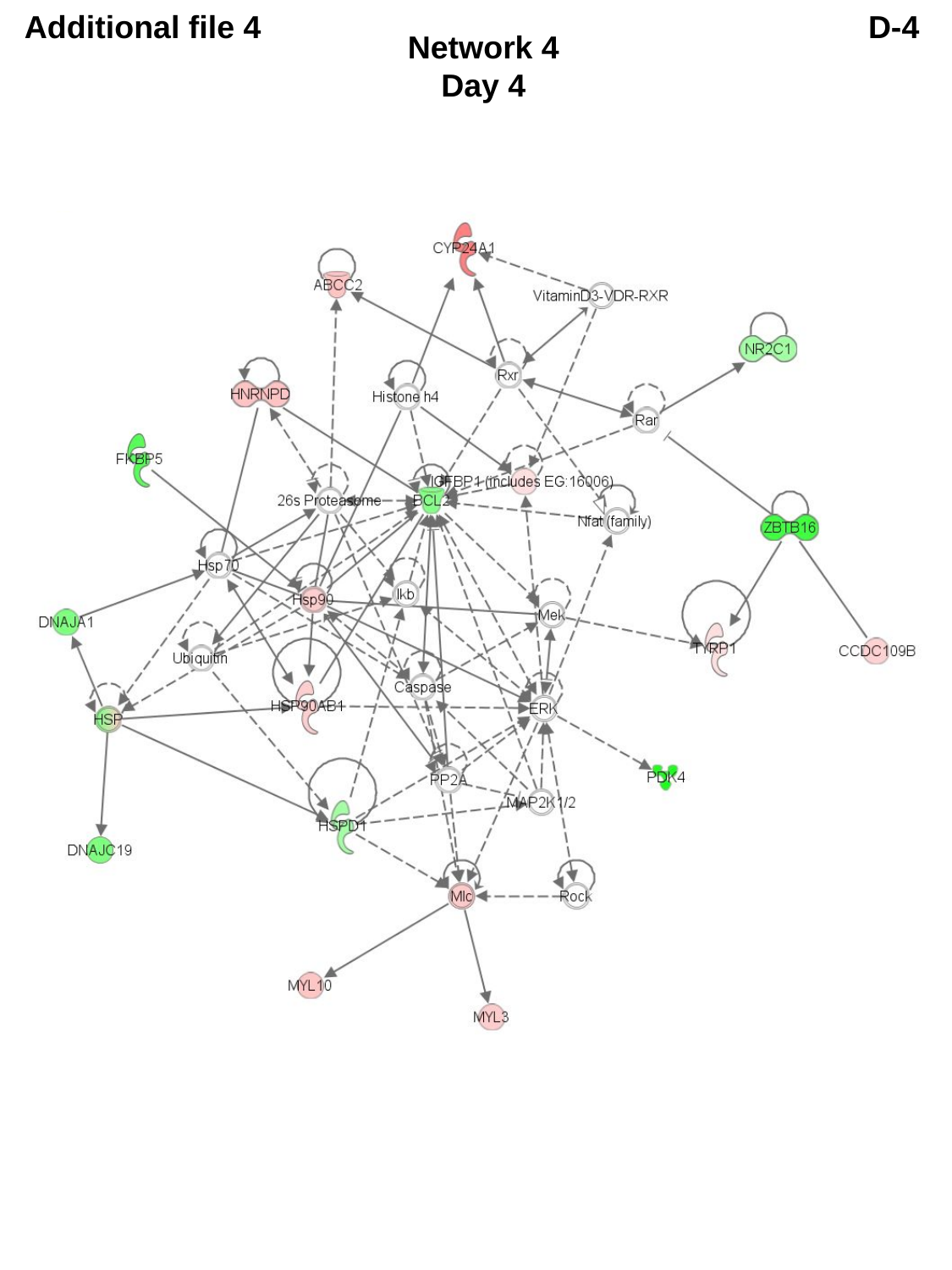

Additional file 4
D-4
Network 4
Day 4
