# Supplementary material for: Genome-wide host responses against infectious laryngotracheitis virus vaccine infection in chicken embryo lung cells
Source: BMC Genomics. 2012 Apr 24;13:143. doi: 10.1186/1471-2164-13-143 (PMC3353197; doi:10.1186/1471-2164-13-143)
Supplement: Additional file 5 — The 21 genes showing a similar expression pattern in both virulent strain and vaccine ILTV infection. [file 1471-2164-13-143-S5.DOC]

**Additional file 5. The 21 genes showing a similar expression pattern in both virulent strain and vaccine ILTV infection**

| **GenBank** | **Symbol** | **Name** |
| --- | --- | --- |
| AB055783 | CENPH | centromere protein H |
| AB105812 | GEM | GTP binding protein overexpressed in skeletal muscle |
| AF051399 | FBLN1 | fibulin 1 |
| AJ309540 | IL6 | interleukin 6 |
| AJ719326 | MMP7 | matrix metalloproteinase 7 |
| AJ719339 | NASP | nuclear autoantigenic sperm protein (histone-binding) |
| AJ720813 | HNRNPD | heterogeneous nuclear ribonucleoprotein D or AU-rich element RNA binding protein 1, 37kDa |
| AY265159 | LHFPL5 | lipoma HMGIC fusion partner-like 5 |
| BX930381 | EMP1 | epithelial membrane protein 1 |
| BX931297 | CYTL1 | cytokine-like 1 |
| BX931418 | IKBIP | inhibitor of NFκB kinase subunit beta interacting protein |
| BX932426 | LRRC6 | leucine rich repeat containing 6 |
| BX932427 | BATF3 | basic leucine zipper transcription factor, ATF-like 3 |
| BX933215 | SOCS1 | suppressor of cytokine signaling 1 |
| BX933888 | C1QTNF3 | C1q and tumor necrosis factor related protein 3 |
| CR353484 | C9orf91 | chromosome 9 open reading frame 91 |
| CR387407 | C2orf77 | chromosome 2 open reading frame 77 |
| M61145 | PRNP | prion protein p27-30 |
| M80584 | LUM | lumican |
| U34977 | FMOD | fibromodulin |
| Y09235 | GLRX | glutaredoxin or thioltransferase |
